# Studying Autism Spectrum Disorder with Structural and Diffusion Magnetic Resonance Imaging: A Survey

**DOI:** 10.3389/fnhum.2016.00211

**Published:** 2016-05-11

**Authors:** Marwa M. T. Ismail, Robert S. Keynton, Mahmoud M. M. O. Mostapha, Ahmed H. ElTanboly, Manuel F. Casanova, Georgy L. Gimel'farb, Ayman El-Baz

**Affiliations:** ^1^BioImaging Laboratory, Department of Bioengineering, University of LouisvilleLouisville, KY, USA; ^2^Departments of Pediatrics and Biomedical Sciences, University of South CarolinaColumbia, SC, USA; ^3^Department of Computer Science, University of AucklandAuckland, New Zealand

**Keywords:** autism, structural MRI, diffusion tensor MRI, multi-modal approaches, life stages

## Abstract

Magnetic resonance imaging (MRI) modalities have emerged as powerful means that facilitate non-invasive clinical diagnostics of various diseases and abnormalities since their inception in the 1980s. Multiple MRI modalities, such as different types of the sMRI and DTI, have been employed to investigate facets of ASD in order to better understand this complex syndrome. This paper reviews recent applications of structural magnetic resonance imaging (sMRI) and diffusion tensor imaging (DTI), to study autism spectrum disorder (ASD). Main reported findings are sometimes contradictory due to different age ranges, hardware protocols, population types, numbers of participants, and image analysis parameters. The primary anatomical structures, such as amygdalae, cerebrum, and cerebellum, associated with clinical-pathological correlates of ASD are highlighted through successive life stages, from infancy to adulthood. This survey demonstrates the absence of consistent pathology in the brains of autistic children and lack of research investigations in patients under 2 years of age in the literature. The known publications also emphasize advances in data acquisition and analysis, as well as significance of multimodal approaches that combine resting-state, task-evoked, and sMRI measures. Initial results obtained with the sMRI and DTI show good promise toward the early and non-invasive ASD diagnostics.

## 1. Introduction

The term “autism spectrum disorder” (ASD) refers to a collection of neuro-developmental disorders that affect linguistic, behavioral, and social skills. Autism has many symptoms, most prominently, social impairment and repetitive behaviors. The most severe form of the ASDs is autistic disorder (AD), while milder forms include Asperger syndrome (ASP), childhood disintegrative disorder, and not-otherwise-specified pervasive developmental disorder (NPDD). By some estimates, the ASD affects 1 out of 68 eight years of age, with males being four times more likely to develop it than females. About 30% of children with ASD have epilepsy at later stages NIMH (2015). Autism is typically diagnosed at the age of 3; however, some characteristics can sometimes be observed at around 12 months of age. What causes autism is yet unknown; however, it is mainly believed that genetic and environmental factors in complex combinations are responsible NIMH (2015).

After the advent in the nineteen eighties, MRI soon became one of the most promising non-invasive modalities for visualization and diagnostics of ASD-related abnormalities. Along with its main advantage of no exposure to radiation, high contrast and spatial resolution, the recent advances to MRI modalities have notably increased diagnostic certainty. The modalities, being most helpful for studying ASD, include structural MRI (sMRI; Damasio and Maurer, 1978; Bauman and Kemper, 1985; Gaffney et al., 1987a,b; Courchesne et al., 1988; Gaffney et al., 1988; Horwitz et al., 1988; Minshew and Payton, 1988; Ritvo and Garber, 1988; Gaffney et al., 1989; Garber et al., 1989; Murakami et al., 1989), and diffusion tensor imaging (DTI; Gropman et al., 2010; Mori and Tournier, 2013).

In applications to ASD, sMRI helps in investigating structural brain changes in autistic subjects. Many scan sequences of the sMRI are volumetric, i.e., allow for measuring specific brain structures to calculate tissue volumes. In spite of diagnostic abilities found for the sMRI, the earlier published results obtained for a limited number of subjects were often contradictory. Specific pulse sequences employed in the sMRI help to reveal different properties of normal and abnormal brain tissues. Modifying the pulse sequence parameters, such as repetition time (TR) and echo time (TE), may emphasize the contrast between gray matter (GM) and white matter (WM), e.g., in the T1-weighted sMRI with short TR and TE, or brain tissue and cerebrospinal fluid (CSF), e.g., in the T2-weighted sMRI with long TR and TE.

The recent DTI characterizes three-dimensional (3D) diffusion of water molecules in a biological tissue Basser et al. (1994a,b). The DTI has a wide range of clinical applications. In particular, it is used to examine normative white matter (WM) development, neurodevelopmental disorders, and neurodegenerative disorders, e.g., autism, and amyotrophic lateral sclerosis Dong et al. (2004).

In neurological studies, where each patient's status can be assessed, the chosen imaging modality should be able to clearly demonstrate the abnormalities. The conventional MRI lacks sensitivity in distinguishing the abnormalities on an individual-subject basis Mori and Tournier (2013), whereas the DTI has the potential to reveal such abnormalities. This new information comes from better image contrast, more detailed WM morphology, refined anatomical locations, and more accurate connectivity analysis Mori and Tournier (2013). Additional DTI offers many contrast-related measurements, including the widely used fractional anisotropy (FA) and estimates of shapes and sizes of specific WM tracts, i.e., WM morphology Mori and Tournier (2013). Moreover, DTI provides superior anatomical information for clearer identification of areas with WM abnormalities Gropman et al. (2010). It also provides unique brain connectivity measurements via 3D fiber reconstruction, e.g., tractography, Basser et al. (2000).

This survey presents applications of the sMRI and DTI to study the ASD (mostly, for the last two decades) in order to outline the most important findings with these modalities across all life stages. This provides a comprehensive study unlike the recent surveys that either focus on one modality, Palmen and van Engeland (2004); Blackmon (2015); Conti et al. (2015); Rane et al. (2015), or address a specific life stage, Conti et al. (2015); Zeglam et al. (2015). Moreover, this survey highlights the methodologies conducted in each study and categorizes them with respect to the approach (e.g., whether they are volumetric-based, or surface-based in sMRI), and user's intervention (manual, automated, semi-automated).

The survey is structured as follows: Sections 2, 3 below address the use of the sMRI and DTI, respectively. Findings in the reviewed publications emphasize benefits for studies of the ASD, as well as other medical abnormalities, by acquiring and analyzing complementary multi-modality data.

## 2. Studying ASD with sMRI

The earlier findings of the 1980s–1990s Damasio and Maurer (1978); Bauman and Kemper (1985); Gaffney et al. (1987a,b); Courchesne et al. (1988); Gaffney et al. (1988); Horwitz et al. (1988); Minshew and Payton (1988); Ritvo and Garber (1988); Gaffney et al. (1989); Garber et al. (1989); Murakami et al. (1989); Nowell et al. (1990); Hsu et al. (1991); Zola-Morgan et al. (1991); Garber and Ritvo (1992); George et al. (1992); Hashimoto et al. (1992, 1993); Holttum et al. (1992); Kleiman et al. (1992); Piven et al. (1992); Bailey et al. (1993); Courchesne et al. (1993, 1994a,b); Adolphs et al. (1994); Bachevalier (1994); Bauman and Kemper (1994); Minshew and Dombrowski (1994); Egaas et al. (1995); Hashimoto et al. (1995); Piven et al. (1995); Saitoh et al. (1995); Zilbovicius et al. (1995); Schaefer et al. (1996); Giedd et al. (1996); Piven et al. (1996); Siegel et al. (1996); Ciesielski et al. (1997); Elia et al. (1997); Lainhart et al. (1997); Piven et al. (1997); Bailey et al. (1998); Dawson et al. (1998); Piven et al. (1998); Abell et al. (1999); Aylward et al. (1999); Courchesne et al. (1999); Fombonne et al. (1999); Gillberg (1999); Levitt et al. (1999); Manes et al. (1999); Sears et al. (1999); Townsend et al. (1999) have many contradictions. However, the much better spatial resolution and contrast of the recent advanced MRI technology made the recent findings of the 2000s–2010s more consistent Brambilla et al. (2003); Stanfield et al. (2008); Chen et al. (2011). This survey focuses on the latter studies and attempts to classify the reported abnormalities at different life stages for each of the autism-related anatomical structures.

Cross-sectional or longitudinal sMRI scans, collected at different time instants for the same individual, are studied by either region-of-interest (ROI) based volumetry, or surface-based morphometry (abbreviated as RBV, and SBM respectively). The ***RBV*** usually focuses on the total volume or area measures for a chosen region, but requires manual intervention by experts to delineate it. This age-sensitive and time consuming process depends on level of automation, yet is powerful in a statistical sense. A method that is correlated to RBV and is known as voxel-based morphometry, ***VBM***, targets tissue density, e.g., relative GM concentration, or volume, e.g., regional volume differences of a certain tissue. The ***SBM*** addresses topological shape features, like surface curvature and folding degree, that cannot be obtained directly using the RBV on a brain sMRI. The SBM is applied mostly to the cerebral cortex, along with its lobes and gyrification patterns.

The ASD studies with the sMRI, including data processing tools and main abnormalities found in brain structures are considered below. Abnormalities found in the cerebral cortex; posterior fossa (vermis, brain stem, and cerebellum); corpus callossum; amygdalae; hippocampus, and thalamus, are addressed at four main life stages: infancy (0–2 years); childhood (3–11 years); adolescence (12–18 years), and adulthood (above 18 years). The measurements include also the total brain volume and head circumference.

### 2.1. Studying ASD impacts on anatomical structures with sMRI

#### 2.1.1. Cerebral cortex

This uppermost brain layer that plays a leading role in human intelligence and perception has been the focus of plenty of sMRI studies on ASD. Changes of cortical thickness and gyrification patterns were analyzed with the SBM, whereas the RBV and VBM measured regional differences in volumes and densities of the cerebral GM and WM.

##### 2.1.1.1. Infancy

According to the longitudinal VBM Hazlett et al. (2005) on 51 autistic children and 25 controls (aged 18–35 months), both the cerebral GM and WM volumes increase in autistic brains. The longitudinal RBV on children aged 1.5 to 5 years Schumann et al. (2010) revealed no changes in the occipital lobe. On the other hand, the cerebral GM volumes were significantly enlarged in the frontal, temporal, and parietal lobes, and in the cingulate gyrus (located in the limbic lobe) of autistic toddlers (aged about 2.5 years). Additionally, there were more abnormal growth profiles for females. The RBV in Hazlett et al. (2012) monitored the total brain volume changes for children aged 6 months to compare high risk infants to low risk ones with no autistic family members. The fact that cerebrum or lateral ventricle volumes had no significant difference for both groups confirmed earlier findings that the brain enlargement is a postnatal event, occurring around 12 months of age. Another extended longitudinal study Shen et al. (2013) pursued the goal of identifying 6–9-month-old infants who might later develop the ASD. It was revealed that the ASD causes an extra-axial fluid and is characterized by excessive CSF over the frontal lobes at 6–9 months of age, which persists at 12–15 and 18–24 months. This leads to large total cerebral volumes that tend to increase with age at a higher rate in males, than females. This finding contradicts the earlier one Schumann et al. (2010), which showed more enlargement in females. This inconsistency might be caused by manual segmentation, along with the younger ages and the 20% smaller number of participants in Shen et al. (2013).

##### 2.1.1.2. Childhood

Volumetric, voxel-wise, and thickness changes in the cerebral cortex in children with autism have been extensively studied in the literature. In particular, the autistic children aged 2–3 years had 18% and 12% larger cerebral cortical WM and GM volumes, respectively, than the control ones, while the older children did not demonstrate such enlargement Courchesne et al. (2001). The increased frontal and temporal GM and frontal and parietal WM volumes at such a young age (2–4 years) was confirmed in Carper et al. (2002). The increased cerebral volumes in autistic children aged around 4 years and 6 years were found also in Sparks et al. (2002) and Akshoomoff et al. (2004), respectively. The latter study reported the increased total cerebral volume, as well as the increased WM and GM volumes for children with both low-functioning autism (LFA) and high-functioning autism (HFA), the total cerebral volume being significantly larger in the LFA group.

The VBM of the GM and WM densities on autistic children around 9 years of age Boddaert et al. (2004) has found a significant decrease in the GM density in the superior temporal sulcus and a decrease in the WM density in the right temporal pole. This finding was supported by McAlonan et al. (2005) where the GM density was found to significantly decrease in the frontostriatal and parietal networks, as well as in the ventral and superior temporal gyrus in autistic children aged around 12 years. The RBV revealed the decreased GM volume in the right lateral orbitofrontal cortex in 10-year-old boys in Girgis et al. (2007) and the decreased GM volumes in the parietal, left temporal, and left occipital lobes bilaterally in Brun et al. (2009). The left and right frontal lobes of the autistic boys had enlarged 3.6 and 5.1%, respectively, while all the other lobes grew more significantly.

The cerebral cortices of children with autism were often studied with the SBM as well. The atlas-based SBM in Hardan et al. (2006b) monitored the cortical thickness changes in autistic brains of children aged 10 years. The frontal or occipital lobes did not change, whereas the temporal and parietal lobes had most prominent increases of the total cerebral sulcal and gyral thicknesses. According to the subsequent longitudinal SBM Hardan et al. (2009) on 10-year old autistic children, with a follow-up scan 2 years later, the cortical thickness in autistic subjects has decreased in the frontal, temporal, and occipital lobes, comparing to controls. Also, the SBM Jiao et al. (2010) on 9-year-old children with autism showed the decreased thickness in the right entorhinal, right lateral orbitofrontal, left lateral orbitofrontal, right medial orbitofrontal, left medial orbitofrontal cortex, and right pars triangularis. However, the thickness also increased in the left caudal anterior cingulate cortex and left frontal pole. Significant bilateral differences in sulcal depth in restricted portions of the anterior-insula, frontal-operculum, and in tempoparietal junction, were found in Dierker et al. (2015). The study by Gori et al. (2015) on 4-year-old males was based on extracting features from GM, WM, and CSF to classify autistic and control brains. Only GM features in different subregions showed a classification performance that reached up to 80%.

##### 2.1.1.3. Adolescence

Volumetric and voxel-wise cerebral changes have been investigated in a number of publications. The VBM in Waiter et al. (2004), for example, revealed the increased GM volume in the cortical lobes of 15-year-old autistic males, namely, in the right fusiform gyrus, right temporal and occipital region, and left frontal pole. This work was extended in Waiter et al. (2005) to investigate changes of the WM volume in 15-year-old autistic males and found that the WM volume decreases at the left middle temporal, right middle frontal, and left superior frontal gyri. The VBM in Kwon et al. (2004) conducted on subjects with the HFA and ASP showed the lower cortical GM density in the right inferior temporal gyrus, entorhinal cortex, and right rostral tip of fusiform gyrus. The RBV in Lotspeich et al. (2004) reported a growth of the cerebral GM in both the HFA and LFA subjects, compared to the controls, but only non-significant changes for people with the ASP. The VBM in Chung et al. (2004) on 16-year-old males revealed the lesser WM concentration in the genu, rostrum, and splenium regions in autistic brains, whereas the RBV in Hazlett et al. (2006) on a group of young adolescents has found a larger total cerebral volume of the autistic brains. The lobe volume growth due to the GM volume enlargement was also noticed in the frontal and temporal, but not parietal or occipital lobes. The VBM on autistic and control males around the age of 13 in Bonilha et al. (2008) has found an increase in the GM volume in the parietal lobes, medial and dorsolateral frontal areas, and lateral and medial parts of temporal lobes, as well as a decrease in the WM volume in the frontal, parietal, temporal, and occipital lobes. The longitudinal RBV on 13-year-old adolescents in Hua et al. (2013) has found a decelerated WM growth in the frontal, temporal, parietal, and occipital lobes, together with an abnormally accelerated GM expansion in the putamen and anterior cingulate cortex.

To monitor cortical changes in the adolescent groups due to autism, the SBM on a group of 12-year-old autistic and control adolescents was used in Hardan et al. (2004) to measure changes in the cerebral folding and better investigate the gyrification patterns. As was found, the adolescents had the higher left frontal gyrification index, than the adults, as well as the cortical folding had decreased bilaterally with age in all the autistic subjects, but not in the controls. The SBM on a wide variety of patients including the LFA, HFA, and ASP subjects aged 11–13 years in Nordahl et al. (2007) has found for the LFA subjects prominent shape abnormalities centered on pars opercularis of the inferior frontal gyrus, associated with sulcal depth differences in the anterior insula and frontal operculum. The bilateral shape abnormalities in the HFA group were similar to those of the LFA group, but smaller in size and centered more posteriorly in and near the parietal operculum and ventral postcentral gyrus. The ASP group had the correlated with age bilateral abnormalities in the intraparietal sulcus. All these cortical shape abnormalities were more pronounced in the children than in the adolescents. Two successive longitudinal studies on the same group of subjects when their mean age was 17.4 and 19 years, respectively, in Wallace et al. (2015) demonstrated an accelerated cortical thinning in the autistic brains with respect to the controls in two areas in the left hemisphere, namely, in the posterior portion of the ventral temporal cortex and the superior parietal cortex. This acceleration has happened only for the older adolescents and young adults of the second study.

##### 2.1.1.4. Adulthood

The RBV on 16 autistic males of the average age of 22 years in Hardan et al. (2001b) revealed the larger mean cerebral and third ventricle volumes in the autistic subjects rather than in the controls. The VBM in Rojas et al. (2006) gave the larger GM volume in the medial frontal gyri, left precentral gyrus, right postcentral gyrus, and right fusiform gyrus. The more recent VBM Toal et al. (2010) showed the decreased GM volume in the medial, temporal, and fusiform regions. The SBM in Hardan et al. (2004) explored changes of the cerebral folding in 27-year-old males. While the left frontal gyrification index had no changes, the cortical folding decreased in autistic adults, compared to children and adolescents. The SBM in Hadjikhani et al. (2006) on the autistic, ASP, and NPDD subjects aged around 33 years has shown the decreased thickness in the inferior frontal gyrus, pars opercularis, inferior parietal lobule, superior temporal sulcus, precentral and postcentral gyrus, inferior occipital gyrus, prefrontal cortex, anterior cingulate, medial parietal cortex, supramarginal gyrus, and middle and inferior temporal cortex. The SBM in Hyde et al. (2010) on 15 autistic 22.7-year-old (on average) males has shown that the thickness increases in the frontal, temporal, occipital, cingulate, and parietal gyrus, as well as in the fusiform gyri, but decreases in the pre- and post-central gyri and para-central gyrus.

##### Conclusions

According to the above studies, the cerebral cortex starts changing in the autistic brains at the age of around 12 months, and no significant differences could be monitored earlier, except of the recent findings in Shen et al. (2013). The cerebrum changes of an autistic brain at such an early age include the age-proportional enlargements of the lobes and increases of the cortical WM and GM. The growing cerebral volume, including the GM and WM, in early childhood indicates simultaneously the decreasing GM and WM density. Also, children with autism demonstrate significant changes in the cortical thickness. These volumetric and thickness differences continue to increase at the later stages of life.

#### 2.1.2. Posterior cranial fossa (cerebellum, vermis, and brain stem)

Posterior cranial fossa has also been investigated in the literature for any correlates with ASD at different life stages.

##### 2.1.2.1. Childhood

Area measurements on 22 autistic children in Elia et al. (2000) showed no abnormalities in the total vermis, vermis lobules VI-VII, pons, and midbrain, which could be related to autism. However, the decreased WM density in the cerebellum of children with autism was reported in McAlonan et al. (2005) and Boddaert et al. (2004), and the larger by 39% cerebellar WM volume was found in Courchesne et al. (2001) for 2–3-year-old children with autism compared to controls. Autistic boys had the lesser GM and smaller GM-to-WM ratios and vermian lobules than the normal ones. The reduced cross-sectional areas of the vermis lobules VI-VII in autistic children were also reported in Courchesne et al. (2001), Kaufmann et al. (2003), and Carper and Courchesne (2000). The larger cerebellar WM and GM volumes and increased area of the anterior and posterior cerebellar vermis were found in the 6-year-old children with the LFA, HFA, and NPDD in Akshoomoff et al. (2004).

Multiple brain stem volume studies gave contradicting conclusions. The early areal measurements in Gaffney et al. (1988) and Ciesielski et al. (1997) claimed reduced brain stem size, whereas the subsequent works, such as Garber and Ritvo (1992), Piven et al. (1992), Kleiman et al. (1992), Hsu et al. (1991), and Elia et al. (2000) found no significant differences between the autistic and control groups. No differences in the brain stem volumes for these groups were found also in Hardan et al. (2001a) and Herbert et al. (2003). However, the recent study Jou et al. (2013) of 10-year-old children, with a follow-up after 2 years, came up with different findings: the brain stem volume was stable in the controls over the 2-year period, but the increased GM volume of an autistic brain implied the larger entire brain stem volume, so that the autistic brain volumes eventually became comparable to those of the 15-year old controls.

##### 2.1.2.2. Adolescence and adulthood

An increase in the GM volume in the cerebellum in Bonilha et al. (2008) is consistent with the studies of children. The cerebellar volumes of autistic children and adolescents are also consistent with those of adults. As shown in Hardan et al. (2001a) on 22-year-old subjects, the total cerebellar volume and cerebellar hemispheres are larger in the autistic group both with and without correction by the total brain volume. But the volumetric and area measurements of the vermis and brain stem did not differ significantly between the autistic and control groups. Also, the lower GM density in the frontostriatal and cerebellar regions, along with the widespread WM differences, had been reported in McAlonan et al. (2002) and the lower brain stem and total brain stem GM volumes in the autistic adults have been found in Jou et al. (2009).

##### Conclusions

The posterior fossa structures are significantly affected with the ASD from an early age of 2 years and during all the subsequent life stages. Some inconsistencies in the brain stem abnormalities found might be caused by wide age and gender differences of the participants.

#### 2.1.3. Amygdalae

Amygdalae have also been investigated in efforts to find any correlates with the ASD.

##### 2.1.3.1. Infancy

To our knowledge, the amygdalar changes in the infancy period are not studied yet, and the youngest subjects in such studies are around 3 years old.

##### 2.1.3.2. Childhood

The RBV on 29 autistic and 26 control subjects (the average age of 3.9 years) in Sparks et al. (2002) has shown that in the autistic subjects the amygdalae are enlarged proportionally to the overall increase of the entire cerebral volume. The more recent volumetry Munson et al. (2006) on a larger group of the 45 autistic subjects suggested the enlargement of only the right amygdalar volume at the ages of 3–4 years. The longitudinal RBV in Nordahl et al. (2012), which was performed in order to precisely define the age at which the amygdalae begin to enlarge, started with the 85 autistic subjects aged 37 months on average, and had the follow-up scan 1 year later on 45 of these autistic subjects. That enlarging the amygdalar volume was found in both the cases, although with a higher rate for the latter group, confirms that this enlargement is present at such an early age of around 3 years. The volumetry on an older population (7.5–12.5 years) in Schumann et al. (2004) demonstrated that both the right and left amygdalae volumes were enlarged. However, the longitudinal RBV in Barnea-Goraly et al. (2014), which involved 15 autistic subjects aged 10.6 years on average and their follow-up scan after reaching adolescence, has found no significant difference between the left, right, or total amygdalar volumes for the autistic and control subjects. This contradiction to all other studies might be caused by the relatively small population used.

##### 2.1.3.3. Adolescence

No significant difference in left, right, or total amygdalae volumes in the adolescents was found in various studies, e.g., Schumann et al. (2004) and Barnea-Goraly et al. (2014).

##### 2.1.3.4. Adulthood

The RBV in Rojas et al. (2004) explored the hypothesis that parents of autistic children would show similar structural changes. This study was conducted in part on the amygdalar volumes of 15 autistic subjects (the average age of 30.3 years), 17 controls (the average age of 43.6 years), and 17 parents of autistic children. The amygdalae were smaller in the autistic group than in the other two groups.

##### Conclusions

The amygdalar volumes generally increase in autistic brains at early stage of life, with the rate of increase being proportional to the age (the known studies started from the 3-year-old subjects). By adolescence, no significant differences in this structure could be found. However, at the subsequent life stages its volume decreases in autistic brains.

#### 2.1.4. Hippocampus

Hippocampus also proved to have some correlates with the ASD from a young age.

##### 2.1.4.1. Infancy

Similarly to Section 2.1.3 above, no hippocampal changes have been explored at this period, with the youngest subjects age having been around 2.5–3 years.

##### 2.1.4.2. Childhood

The enlarged hippocampus was found in autistic children at the ages of both 3–4 Sparks et al. (2002) and 7.5–12.5 years Schumann et al. (2004), especially at the HFA group in the latter study. The enlargement only in the right hippocampus in children aged around 10 years was reported in Barnea-Goraly et al. (2014). The earlier study Saitoh et al. (2001) suggested that the area dentata in autistic subjects was significantly smaller than in the normal ones, with the largest deviation at the ages from 29 months to 4 years.

##### 2.1.4.3. Adolescence

The hippocampus was found enlarged in autistic subjects in Schumann et al. (2004); however, this finding differs from the more recent results in Barnea-Goraly et al. (2014), where the right hippocampal volume has increased in the autistic children, but not in the adolescents. In the latter case, it was found that differences between hippocampal volumes in autistic and control brains became insignificant with time.

##### 2.1.4.4. Adulthood

The hippocampus of autistic adults was confirmed to be significantly enlarged Rojas et al. (2004), compared to controls and also to parents of autistic children. The left hippocampus was larger in both the parents of autistic children and the adults with autism, compared to the controls. The VBM in Rojas et al. (2006) has shown an increase in the GM volume of the left hippocampus.

##### Conclusion

In autism, the hippocampus is enlarged at all ages.

#### 2.1.5. Corpus callosum

Corpus callosum is found to be correlated with the ASD. In particular, the two successive longitudinal RBV studies Frazier et al. (2012) on 19 subjects with autism and 4 subjects with NPDD (first, at the age of 10.6 years and then 13.1 years on average) have found persistent reductions in the total corpus callosum volumes in the autistic subjects compared to the healthy controls. Only the size of rostral body subdivision has normalized over time. The centerline length of the corpus callosum was significantly reduced in young adults with autism compared to controls Elnakib et al. (2011).

##### 2.1.5.1. Conclusion

The corpus callosum is reduced in size in the ASD.

#### 2.1.6. Thalamus, caudate, and putamen

The GM volume decreases in the right thalamus of adolescent males with autism and the thalamic volume decreases in adults with autism compared to controls, as was found, respectively, in Waiter et al. (2004) and Tsatsanis et al. (2003). However, no volume differences of the basal ganglia, caudate, or putamen at all ages have been found in Hardan et al. (2003). The more recent RBV by the same research group Hardan et al. (2006a) found no differences in both the right and left thalamic nuclei between the 19-year-old, on average, control and autistic groups. Studying the older adults (aged 28 years on average) showed that the right caudate nucleus has a larger volume in the autistic brain Haznedar et al. (2006).

#### 2.1.7. Total brain volume and head circumference

Changes in the total brain volume and head circumference have been associated with the ASD in several publications.

##### 2.1.7.1. Infancy

As shown in Hazlett et al. (2005) for 18–35 month-old infants, the total brain volume increases in the group with autism and their normal at birth head circumference becomes significantly larger around 12 months of age compared to the healthy group. This finding was confirmed in Hazlett et al. (2012), showing no significant difference in the head circumference at 6 months of age in infants with high risk of autism.

##### 2.1.7.2. Childhood

The head circumference grows also in children with autism. The increased brain volume and head circumference in a large group of 10-year-old children with autism have been confirmed in Aylward et al. (2002) and supported in other studies, e.g., in Herbert et al. (2003) on 9-year-old autistic males and in Carper et al. (2002). However, a more recent publication Hardan et al. (2006b) reported insignificant increases in the total brain volumes for children with autism (this contradiction to the above findings might be due to a small number of participants). The later stages of life show no difference between the autistic and neurotypical groups in the total brain volumes, probably because the growth rate of the normal brains increases at these stages.

### 2.2. Summary

Tables 1–4 summarize, in accordance with the life stage, basic current results of studying the ASD with the sMRI.

**Table 1:** **sMRI-based studying of the ASD at infancy**.

**Rf**	**Autistic group**			**Control group**			**sMRI**	**Brain regions**	**Data analysis**	**Findings**
	**Age, mo**	**IQ**	**Size**	**Age, mo**	**IQ**	**Size**				
Hazlett et al., 2005	18–35	45–63	46 m; 5 f	18–35	89–127 TD; 49–68 DD	10 m, 4 f TD; 6 m, 5 f DD	1.5T; 1.5 mm	Cerebral GM, WM; cerebellar GM, WM	RBV; NLMM; BRAINS2 + EMS to segment tissues	Increase in cerebral and no change in cerebellar GM and WM volumes at 24 mo of age
Schumann et al., 2010	22–67 m; 26–58 f	FSIQ: 39–75 m; 34–80 f	32 m; 9 f	12–63 m; 12–61 f	FSIQ: 95–127 m; 101–131 f	32 m; 12 f	1.5 T; 1.5 mm	Cerebral GM, WM	RBV; SPSS14.0; SPSS16.0; ANCOVA + semi-automatic GWseg segmentation	Significant enlargement in cerebral WM and GM; notably, in frontal, temporal, and cingulate cortices by 30 mo of age (more abnormal growth of f than m)
Hazlett et al., 2012	6–7	–	61 m; 37 f	6–7	–	21 m; 15 f	3 T; 1 mm	Cerebrum, cerebellum	RBV; ITK-SNAP; GLM + AutoSeg, ANOVA; HeadCirc	No significant difference for HC, cerebral cortex, cerebellum, or lateral ventricle volumes; unchanged TBV
Shen et al., 2013	6–9 t1; 13–14 t2; 19–21 t3	–	22 m; 11 f	6–10 t1; 14–15 t2; 21–22 t3	–	15 m; 7 f	3 T; 1 mm	Cerebrum	RBV; LMM; manual segmentation + LS	Excessive CSF over frontal lobes at ages 6–9 mo; large total cerebral volumes at 12–15 mo, 18–24 mo, with higher rate of enlargement in m than f

*Additional abbreviations: f, females; m, males; mo, month; Rf, reference*;

*T, MRI magnetic field strength (tesla); GLM, general linear models; HC, head circumference*;

*ANOVA, analysis of variances; ANCOVA, analysis of covariances; DD, developmental delay*;

*LMM, linear mixed models; LS, longitudinal segmentation; t1, t2, t3, time periods during longitudinal studies*;

*NLMM, non-linear mixed models; TBV, total brain volume; TD, typical development*;

*AutoSeg, BRAINS2, EMS, GWseg, ITK-SNAP, HeadCirc, SPSS14.0, SPSS16.0, data processing packages*.

**Table 2:** **sMRI-based studying of the ASD at childhood**.

**Rf**	**Autistic group**			**Control group**			**sMRI**	**Brain regions**	**Data analysis**	**Findings**
	**Age, y**	**IQ**	**Size**	**Age, y**	**IQ**	**Size**				
Elia et al., 2000	5–17	<70	22 m	5–15	VIQ: 86–132	11 m	0.5 T; 5 mm	Midsagittal area of cerebrum corpus callosum, midbrain cerebellar vermis and vermian lobules	Area measurements; *t*-test; regression analysis	Negative correlation between midsagittal cerebrum area and age in patients with AD; no abnormalities in total vermis, vermis lobules VI-VII, pons, midbrain, and cerebrum areas; no anatomical abnormalities in brain stem
Carper and Courchesne, 2000	4–7	57–102	42 m	4–8	102–126	29 m	1.5 T; 3–4 mm	Vermis lobules, cerebellum frontal lobes	Area measurements; RBV; SEGMENT	Reduced vermis lobules VI-VII areas; no abnormalities in frontal lobe volumes; inverse correlation between lobules areas and frontal cortex volumes
Courchesne et al., 2001	2–16	36–122	60 m	2–16	90–140	52 m	1.5 T; 3–4 mm	Vermis, cerebellum	Area measurements; RBV; SPSS	Larger cerebellar WM and brain volumes in 2−4y-old autistic patients; reduced vermis lobules VI-VII cross-sectional areas
Saitoh et al., 2001	2–42	VIQ: 41–135	52 m; 7 f	2–43	VIQ: 88–150	40 m; 11 f	1.5 T; 5 mm	Hippocampus area dentata and combined area of subiculum	Area measurements; ANOVA; tracing anatomical landmarks	Significantly smaller than normal area dentata in AD subjects; the largest deviation from normal in 29 mo–4 y
Carper et al., 2002	4–8	>70 in 26 m	38 m	2–12	>80	39 m	1.5 T; 3 mm	WM and GM volumes	RBV; SPSS	Increased frontal and parietal WM and frontal and temporal GM volumes in 2–4 y-old AD subjects
Aylward et al., 2002	9–29	87–118	58 m; 9 f	9–29	95–120	76 m; 7 f	1.5 T; 1.5 mm	TBV; HC	RBV; MEASURE	Increased TBV and HC in 8–12 y-old AD subjects
Sparks et al., 2002	AD: 3–5; NPDD: 4–5	<80	AD: 26 m; 3 f, NPDD: 12 m; 4 f	TD: 3–6, DD: 3–6	Normal	TD: 18 m; 8 f, DD: 6 m; 8 f	1.5 T; 1.5−2 mm	Cerebrum, cerebellum, amygdala, hippocampus	RBV; SPSS; ANCOVA	Increased TBV and enlarged amygdala, cerebellar volume, and hippocampus in AD subjects
Kaufmann et al., 2003	5–9	52–81	10 m	6–10	112–130	22 m	1.5 T; 3 mm	Cerebellar vermis: anterior vermis lobules I−V, posterior superior vermis VI−VII, posterior inferior vermis VIII−X	Area measurements; manual segmentation; BrainImage	Reduced in volume vermian lobules VI−VII
Boddaert et al., 2004	7–15	21–64	16 m; 5 f	7–15	–	7 m; 5 f	1.5 T; 1.2 mm	GM, WM regional density	VBA; ANCOVA; SPM99	Significantly decreased GM concentration in temporal sulcus; decreased WM concentration in right temporal pole and cerebellum
Schumann et al., 2004	LFA: 9–16, HFA: 9–16, ASP: 10–16	LFA: 56, HFA: 91, ASP: 106	LFA: 19 m, HFA: 27 m, ASP: 25 m	10–16	104–126	27 m	1.5 T; 1.5 mm	Amygdala, hippocampus	RBV; SPSS; ANOVA; BrainImage5.x	7.5–12.5 y: larger right and left amygdala volumes, enlarged hippocampus, no change in cerebral volume; 12.75–18.5 y: no change in amygdala
Hardan et al., 2004	13–15	89–121	12 m	11–15	97–123	13 m	1.5 T; 1.5 mm	Cerebral folding (gyrification patterns)	SBM; BRAINS2	Higher left frontal gyrification index in children and adolescents, but not adults; decreased bilaterally with age cortical folding in all AD subjects, but not in controls
Akshoomoff et al., 2004	LFA: 5–8, HFA: 4–10, NPDD: 5–8	LFA: 26–63, HFA: 73–85, NPDD: 72–101	LFA: 30 m, HFA: 12 m, NPDD: 10 m	2–5	86–132	15 m	1.5 T; 3 mm	TBV; GM, WM volumes of cerebrum, cerebellum, and cerebellar vermis	RBV; ANOVA; SEGMENT	LFA: significantly larger brain and cerebral volumes than in controls; all AD groups: higher overall cerebral volume, cerebral GM and WM, cerebellar volume, cerebellar GM and WM, and anterior and posterior cerebellar vermis area
McAlonan et al., 2005	10–14	91–111	16 m; 1 f	10–13	100–128	16 m; 1 f	1.5 T; 3 mm	Regional GM and WM density	VBM; MANCOVA; BAMM for measuring brain volumes	Decreased GM density in frontostriatal and parietal networks and in ventral and superior temporal gyrus; decreased WM density in cerebellum and left internal capsule and fornices; in general, severe reduction in GM and significant increase in CSF in autistic brains
Munson et al., 2006	ASD: 4, NPDD: 4–5	–	ASD: 26 m; 3 f, NPDD: 12 m; 4 f	–	–	–	1.5 T; 1.55 mm	Amygdala, hippocampus	RBV; MEASURE	Larger right amygdalar volumes at ages 3–4, but not left amygdalar, hippocampal, or total cerebral volumes
Hardan et al., 2006b	8–13	64–128	17 m	9–13	91–130	14 m	1.5 T; 1.5 mm	Cortical thickness	SBM; BRAINS	Most prominent changes in temporal and parietal lobes: increased total cerebral sulcal and gyral thicknesses; no changes in frontal and occipital lobes
Girgis et al., 2007	8–13	77–109	11 m	9–12	91–131	18 m	1.5 T; 1.5 mm	TBV	RBV; BRAINS2	Decreased GM in right lateral orbitofrontal cortex
Nordahl et al., 2007	LFA: 10–16, HFA: 8–14, ASP: 9–16	LFA: 46–66, HFA: 73–105, ASP: 80–114	LFA: 17 m, HFA: 14 m, ASP: 15 m	9–14	103–127	29 m	1.5 T; 1.5 mm	Cortex	SBM; Caret5.4; Caret5.5	LFA: prominent shape abnormalities centered on pars opercularis of inferior frontal gyrus, associated with sulcal depth differences in anterior insula and frontal operculum; HFA: bilateral shape abnormalities similar to LFA, but smaller in size and centered more posteriorly in and near parietal operculum and ventral postcentral gyrus; ASP: bilateral abnormalities in intraparietal sulcus correlated with age; all cortical shape abnormalities – more pronounced in children
Brun et al., 2009	6–13	FSIQ: 84–107	24 m	8–13	FSIQ: 94–117	26 m	3 T; 1.2 mm	Cortex	RBV; BAMM	Bilaterally decreased GM volumes in parietal, left temporal, and left occipital lobes; enlarged by 3.6% left, and 5.1% right frontal lobes in AD boys; significantly enlarged all other lobes
Hardan et al., 2009	10–12	FSIQ: 77–111	18 m	10–12	100–126	16 m	1.5 T; 1.5 mm	Cortex, TBV	RBV; longitudinal SBM; MANOVA; BRAINS; BRAINS2	Decreased total GM volume over time, decreased cortical thickness in AD subjects in frontal, temporal, and occipital lobes compared to controls
Jiao et al., 2010	7–11	80–124	19 m; 3 f	8–12	87–129	13 m; 3 f	1.5 T; 2 mm	Cortex	SBM; *t*-test; WEKA; FreeSurfer	Decreased thickness in right entorhinal, right lateral orbitofrontal, left lateral orbitofrontal, right medial orbitofrontal, left medial orbitofrontal cortex, and right pars triangularis; increased thickness in left caudal anterior cingulate cortex and left frontal pole
Nordahl et al., 2012	2–4	DQ: 41–85	ASD: 79 m, NPDD: 6 m	2–4	92–116	47 m	3 T; 1 mm	Amygdala	RBV; longitudinal; ANCOVA; ANALYZE 10.0; BET	Amygdala enlargement at two time points with a higher rate at the second point; total cerebral volume enlargement with the same rate at both the points
Frazier et al., 2012	7–13 t1, 9–16 t2	FSIQ: 75–115	ASD: 19 m, NPDD: 4 m	7–13 t1, 9–16 t2	FSIQ: 103–129	23 m	1.5 T; 1.5 mm	Corpus callossum	RBV; longitudinal; MERM; BRAINS2	Persistent reductions in corpus callossum volume in AD subjects compared to healthy controls; size normalization over time of only rostral body subdivision
Jou et al., 2013	7–17	75–115	23 m	7–17	103–129	23 m	1.5 T; 1.5 mm	Brainstem	RBV; longitudinal; ANOVA; BRAINS2	Stable brain stem volume in controls over the 2-y period; increased GM volume of autistic brains resolutiong in the increased whole brainstem volume, comparable to volumes of 15 y-old controls
Dierker et al., 2015	9–12	FSIQ: 99–124	28 m; 6 f	10–12	FSIQ: 104–126	23 m; 9 f	3 T; 1 mm	Cortex sulci	SBM; *t*-test; ANOVA; Freesurfer5.1	Bilateral significant differences in sulcal depth in restricted portions of anterior-insula and frontal operculum and in tempoparietal junctions
Barnea-Goraly et al., 2014	8–12 t1, 11–15 t2	78–118	15 m	7–13 t1, 9–16 t2	105–129	22 m	1.5 T; 1.5 mm	Amygdala, hippocampus	RBV; longitudinal; SPSS; BrainImage	Normalization of amygdala volumes in late childhood and adolescence; significantly larger right hippocampus in AD children; large volume reductions in right hippocampus of autistic individuals, compared to stable or slightly increased hippocampal volumes of healthy controls
Gori et al., 2015	3–5	70–113	21 m	3–5	74–123	20 m	1.5 T; 1.1 mm	GM, WM, CSF	VBM; SPM; Freesurfer	Altered GM in different brain regions for autistic and control brains, resulting in a classification accuracy of up to 80% between the 2 groups

*Additional abbreviations: f, females; m, males; mo, month; Rf, reference; t1, t2, time periods; y, year*;

*ANOVA, analysis of variances; ANCOVA, analysis of covariances*;

*DD, developmental delay; HC, head circumference*;

*MERM, mixed-effects regression models; TD, typical development; T, MRI magnetic field strength (tesla)*;

*MANOVA, multivariate analysis of variances; MANCOVA, multivariate analysis of covariances*;

*TBV, total brain volume; ANALYZE, AREA, BAMM, BET, BrainImage, Caret, FreeSurfer*,

*MEASURE, SEGMENT, SPM99, SPSS, VBA, WEKA, BRAINS, BRAINS2, data processing packages*.

**Table 3:** **sMRI-based studying of the ASD at adolescence**.

**Rf**	**Autistic group**			**Control group**			**sMRI**	**Brain regions**	**Data analysis**	**Findings**
	**Age, y**	**IQ**	**Size**	**Age, y**	**IQ**	**Size**				
Waiter et al., 2004	13–18	FSIQ: 80–122	16 m	14–17	FSIQ: 81–118	16 m	1.5 T; 1.6 mm	TBV; GM, WM volumes	VBM; ANCOVA; *t*-test; SPM2	Increased GM volume in right fusiform gyrus, right temporal occipital region, and left frontal pole; decreased GM volume in right thalamus
Chung et al., 2004	11–21	–	16 m	14–20	–	12 m	3 T; 1.2 mm	WM density	VBM; GLM; SPM99+ GMM for segmentation	Lesser WM concentration in genu, rostrum, and splenium (also in CC due to hypoplasia, rather than atrophy)
Lotspeich et al., 2004	LFA: 10–14, HFA: 10–16, ASP: 10–15	LFA: 36–56, HFA: 86–124, ASP: 84–124	LFA: 13 m, HFA: 18 m, ASP: 21 m	10–15	101–125	21 m	1.5 T; 1.5 mm	CGM	RBV; ANOVA; BrainImage5.X (semi-automated segmentation)	HFA and LFA: Increased CGM w.r.t. controls (for ASP: intermediate between HFA and controls, yet insignificant)
Kwon et al., 2004	HFA: 11–17, ASP: 11–16	–	HFA: 9 m, ASP: 11 m	11–17	–	13 m	3 T; 1.5 mm	GM density	VBM; *t*-test; SPM99	HFA and ASP: decreased GM density in right inferior temporal gyrus, entorhinal cortex, and right rostral tip of fusiform gyrus
Waiter et al., 2005	13–17	FSIQ: 78–123	15 m	14–17	FSIQ: 81–118	16 m	1.5 T; 1.6 mm	GM and WM volumes, TBV	VBM; ANCOVA; SPM2	Decreased WM volume in CC, left middle temporal, right middle frontal, and left superior frontal gyri
Hazlett et al., 2006	15–24	52–136	23 m	18–26	91–113	15 m	1.5 T; 1.5 mm	CGM and CWM volumes	RBV; RMML + BRAINS2 for tissue classification	Increased total cerebral and GM volume in AD brains with disproportionately increased left-sided GM volume; enlarged volumes of frontal and temporal, but not parietal or occipital lobes
Bonilha et al., 2008	8–16	–	12 m	8–18	–	16 m	2 T; 1 mm	GM, WM of cortical lobes, cerebellum, and claustrum	VBM; *t*-test + SPM5 for tissue segmentation	Increased GM volume in medial and dorsolateral frontal areas; lateral and medial parts of temporal lobes, and cerebellum and claustrum of parietal lobes; decreased WM volume in frontal, parietal, temporal, and occipital lobes
Hua et al., 2013	10–14 t1, 13–17 t2	FSIQ: 75–121	13 m	10–15 t1, 13–18 t2	FSIQ: 105–131	7 m	1.5 T; 1.2 mm	WM, frontal, parietal, temporal, and occipital lobes	RBV; longitudinal; BSE; Brainsuite	Decelerated WM growth in frontal, temporal, parietal, and occipital lobes; abnormally accelerated GM expansion in putamen and anterior cingulate cortex
Wallace et al., 2015	15–20 t1, 17–22 t2	FSIQ: 104–130	17 m	16–19 t1, 18–22 t2	FSIQ: 106–126	18 m	3 T; 1.2 mm	Cortex	Area measurements; longitudinal; LS GLM; FreeSurfer5.1	Accelerated cortical thinning in AD brains w.r.t. controls in two areas of the left hemisphere: the posterior portion of ventral temporal cortex and superior parietal cortex (only in t2 in late adolescents and young adults)

*Additional abbreviations: f, females; m, males; Rf, reference; t1, t2, time periods; w.r.t., with respect to*;

*T, MRI magnetic field strength (tesla); y, year; CGM, cortical GM; CWM, cortical WM; GLM, general linear model*;

*ANOVA, analysis of variances; ANCOVA, analysis of covariances; CC, corpus callossum*;

*GMM, Gaussian mixture model; LS, least squares; RMML, repeated measures mixed model*;

*BrainImage, BRAINS2, Brainsuite, BSE, FreeSurfer, SPM – data processing packages*.

**Table 4:** **sMRI-based studying of the ASD at adulthood**.

**Rf**	**Autistic group**			**Control group**			**sMRI**	**Brain regions**	**Data analysis**	**Findings**
	**Age, y**	**IQ**	**Size**	**Age, y**	**IQ**	**Size**				
Hardan et al., 2001b	12–32	FSIQ: 88–118	16 m	12–32	FSIQ: 87–15	19 m	1.5 T; 5 mm	Third and fourth lateral ventricles, intracranial and cerebral volumes	RBV; ANCOVA; IMAGE1.45	Larger mean cerebral and third ventricles volumes in AD subjects after adjusting for intracranial volume and thus increasing TBV
Hardan et al., 2001a	12–52	FSIQ: 85–115	22 m	13–52	FSIQ: 87–115	22 m	1.5 T; 5 mm	Cerebellum, vermis, brain stem	RBV; area measurements; ANCOVA; IMAGE	Larger total cerebellar volume and cerebellar hemispheres in AD group with and without TBV correction; no significant differences in vermis and brainstem volumes and areas between groups
McAlonan et al., 2002	22–42	79–111	19 m; 2 f	26–40	100–128	22 m; 2 f	1.5 T; 1.5 mm	GM and WM density in cerebellum	VBM; SPSS; SMart toolkit	Decreased GM density in frontostriatal and cerebellar regions; widespread differences in WM
Hardan et al., 2003	9–45	FSIQ: 88–118	38 m; 2 f	10–44	FSIQ: 94–114	39 m; 2 f	1.5 T; 1.5 mm	TBV, caudate	RBV; NIH Image (semi-automated segmentation)	No volume differences of basal ganglia and no difference in caudate and putamen (all ages)
Tsatsanis et al., 2003	11–38	82–141	12 m	11–30	87–138	12 m	1.5 T; 1.2 mm	TBV, thalamus	RBV; ANOVA; ANALYZE + manual segmentation	Significantly smaller mean thalamic volume in AD brains; positive correlation between TBV and thalamus in control group, yet insignificant in AD group (i.e., the absent in autism increase in thalamic volume with increase in TBV suggesting underdeveloped connections between cortical and subcortical regions)
Hardan et al., 2004	19–37	FSIQ: 88–112	18 m	19–28	FSIQ: 99–115	19 m	1.5 T; 1.5 mm	Cerebral folding	SBM; BRAINS2	No changes in left frontal gyrification index; decreased cortical folding in AD adults w.r.t. AD children and adolescents
Rojas et al., 2004	21–39	74–120	13 m; 2 f	41–49	109–135	8 m; 9 f	1.5 T; 1.7 mm	TBV; amygdala; hippocampus	RBV; Statistica 5.3; IDL 5.3	Larger left hippocampus was in both parents of AD children and adults with AD, w.r.t. control subjects; significantly larger hippocampus in AD adults, than in parents of AD children; smaller left amygdala in AD adults w.r.t. other two groups; no TBV differences between all groups
Hadjikhani et al., 2006	21–45	98–128	AD: 8 m, ASP: 4 m, NPDD: 2 m	22–40	105–131	14 m	1.5 T; 1.25 mm	Cortex	SBM	Decreased thickness in inferior frontal gyrus pars opercularis, inferior parietal lobule, superior temporal sulcus, precentral gyrus, postcentral gyrus, inferior occipital gyrus, prefrontal cortex, anterior cingulate, medial parietal cortex, supramarginal gyrus, and middle and inferior temporal cortex
Hardan et al., 2006a	8–45	75–135	38 m; 2 f	9–43	86–121	39 m; 2 f	1.5 T; 3 mm	Thalamus	RBV; NIH Image	No difference between two groups in RBV of right and left thalamic nuclei; no linear relationship between TBV and thalamic volume
Rojas et al., 2006	9–44	FSIQ: 60–133	24 m	8–44	FSIQ: 99–139	23 m	1.5 T; 1.7 mm	Regional GM volume	VBM; longitudinal; ANCOVA; SPM2	Increased GM volume in medial frontal gyri, left precentral gyrus, right postcentral gyrus, right fusiform gyrus, caudate nuclei, and left hippocampus; decreased GM volume in cerebellum
Haznedar et al., 2006	17–55	55–125	AD: 10, ASP: 7; 15 m; 2 f	20–56	88–136	15 m; 2 f	1.5 T; 1.2 mm	Thalamus	RBV; ANOVA; manual segmentation	Larger volumes of right caudate nucleus in AD brains
Hyde et al., 2010	14–33	FSIQ: 89–113	13 m	14–34	FSIQ: 95–119	15 m	3 T; 1.5 mm	Cortical thickness	SBM; GLM; CIVET	Increased thickness in frontal, temporal, occipital, cingulate, and parietal gyrus and fusiform gyri; decreased thickness in pre- and postcentral gyri and paracentral gyrus
Toal et al., 2010	AD: 18–49, ASP: 16–59	AD: 53–133, ASP: 78–141	AD: 21 m; 5 f, ASP: 35 m; 4 f	19–58	74–122	30 m	1.5 T; 1.5 mm	GM and WM volumes	VBM; ANCOVA; SPSS; SPM2	Decreased GM volume in medial temporal, fusiform, and cerebellar regions; decreased WM volume in brain stem portions of cerebellum
Elnakib et al., 2011	19	–	17 m	19	–	17 m	1.5 T; 1.25 mm	CC	Area measurements	Significant reduction in the CC length of AD subjects

*Additional abbreviations: f, females; m, males; mo, month; Rf, reference*;

*T, MRI magnetic field strength (tesla)*;

*CC, corpus callosum; TBV, total brain volume*;

*y, year; w.r.t., with respect to; ANOVA, analysis of variances; ANCOVA, analysis of covariances*;

*ANALYZE, BRAINS, CIVET, IDL, IMAGE, NIH Image, SMart, SPM, SPSS, Statistica, data processing packages*.

## 3. Studying ASD with DTI

DTI characterizes 3D diffusion of water molecules in a biological tissue with DT Basser et al. (1994a,b). The DTI is widely used in clinical applications, e.g., to examine a normative WM development, neurodevelopmental disorders, like ASD, and neurodegenerative disorders, such as amyotrophic lateral sclerosis Dong et al. (2004). For completeness, basic principles of the DTI are reviewed first below.

### 3.1. DTI: Basic concepts

An unconstrained medium, such as the CSF, ensures the isotropic diffusion where the water molecules move in a similar manner in all directions. The isotropic diffusion is typical, e.g., for the brain ventricles, whereas the molecular diffusion in the WM is constrained by spatial orientations of the WM tracts. The molecules can diffuse along a fiber track more freely than across it, so that the diffusion becomes anisotropic with a privileged direction. As a result, water diffusion patterns for the brain tissues provide information about the underlying anatomical structures Mori and Tournier (2013).

Generally, a fiber is oriented arbitrarily and has different diffusion coefficients along different directions.

DT is usually specified with certain parameters at each voxel, that are usually converted into maps of scalar diffusion measurements to facilitate interpreting the voxel-wise DTI data. The most common anisotropy and microstructural measurements include fractional (FA) and relative (RA) anisotropy together with mean (MD), axial (λ_||_), and radial (λ_⊥_) diffusivity.

The orientation-independent **mean diffusivity**, also called the *trace*, measures an overall diffusion in a voxel or region. A slightly different MD definition has been used to measure the diffusion descent in brain ischemia van Gelderen et al. (1994). Since the MD values in the CSF are higher than it is in other types of brain tissues, the MD is recommended for the CSF-related disease studies Narr et al. (2009).

The **fractional anisotropy**, described first in Koay et al. (2006), is the most popular rotationally invariant (i.e., orientation-independent) measure of how isotropic is the voxel- or region-wise diffusion. The FA of a physically realizable diffusion with non-negative eigenvalues ranges from 0 to 1 in the opposite extreme complete isotropic and linear anisotropic cases, respectively. For example, the WM appears whiter due to its higher FA. The reduced FA values usually indicate changes in myelination or degraded axonal structures of the WM Lerner et al. (2014).

The **relative anisotropy** is similar to the FA and takes the range between 0 (the complete isotropy) to $\sqrt{2}$ (the complete, i.e., linear anisotropy). The RA is also defined as the ratio of anisotropic and isotropic parts of the diffusion Le Bihan et al. (2001).

The **axial**, or *parallel* **diffusivity**, λ_||_, measures the diffusion along the principal axis (parallel to axons), whereas the **radial**, or *perpendicular* **diffusivity**, λ_⊥_, averages the diffusion along the two minor axes to measure the degree of restriction due to membranes and other effects.

These two measurements are closely connected to the WM pathology Alexander et al. (2007) and have been used to observe developmental and pathological fiber alterations, e.g., to study dysmyelinating disorders Song et al. (2005).

Main fiber trajectories are extracted from the DTI and visualized using 2D color-coded **fiber orientation maps**. These maps provide unique information, which cannot be obtained with other MRI techniques. In particular, the fiber orientations could classify and stratify specific WM tracts in a host of medical applications that need more anatomical details Alexander et al. (2007).

These 2D color-coded maps present the voxel-wise fiber orientations related to the WM tracts, but cannot reveal 3D WM trajectories and connection patterns Mori and Tournier (2013). The latter are obtained with the computer-aided 3D **WM fiber tractography**, recognizing and tracing WM tracts and their connections with other WM tracts or GM structures. The tractography is either *deterministic*, or *probabilistic* Basser et al. (2000); Nucifora et al. (2007), and constructs, respectively, only one trajectory for each start voxel, or the most probable, or minimum-energy path between two selected voxels or regions Mori and Tournier (2013).

The **deterministic** (or tract propagation) **fiber tractography** is built by extracting fiber orientation and propagating pathway until termination criteria are met Mori and Tournier (2013). Generally, the local voxel-wise fiber orientations are estimated directly from planar diffusion profiles. These estimates fail if the ellipsoid is isotropic or the diffusion profile is planar.

Image noise, patient movements, and other imaging artifacts cause uncertainty in the fiber orientations obtained by the deterministic fiber tractography. The **probabilistic fiber tractography** attempts to increase the confidence Basser and Jones (2002) by estimating probability distributions of all fiber orientations and selecting the most probable orientations. Tracing many different pathways with marginally altered orientations allows for measuring the connection probability and assessing fiber connectivity between different brain regions by a voxel-wise connectivity index Behrens et al. (2003). The main advantage of the probabilistic fiber tractography is its ability to stratify the entire WM tracts. However, its accuracy is limited and depends on the accuracy of the DT and estimated pathway probability distributions. Moreover, the probabilistic tractography cannot differentiate between ante- and retrograde along the fiber's path Basser and Jones (2002).

### 3.2. Studying ASD impacts on anatomical structures with DTI

Recent molecular and functional ASD studies confirmed the vital importance of localizing atypical development and examining neural networks and connectivity of different brain areas Travers et al. (2012). At the molecular level, the postmortem studies of brains of children with ASD have shown more reduced and less compact minicolumns Casanova et al. (2002a,b, 2006). At the functional level, examining brain connectivity with the fMRI revealed how activities of various brain areas are organized. Most of the ASD patients have demonstrated reduced connectivity between the frontal and posterior brain parts during various cognitive tasks and in a resting state Schipul et al. (2011). Therefore, according to both molecular and functional studies, the brain connectivity and the underlying WM tracts might be impaired in these patients.

However, earlier studies had limited abilities to provide sufficient morphological information about these WM tracts and their development in a living human Travers et al. (2012). To overcome this drawback, multiple noninvasive DTI-based studies of both the macro- and microstructure (e.g., axons) of the brain WM tracts were conducted for the last decade to investigate the ASD Travers et al. (2012). In particular, the DTI facilitated examining microstructural properties of the WM circuitry and detecting abnormalities of the WM fiber tract integrity Wolff et al. (2012). Below, the most important current methods of studying the ASD with the DTI (in total, from 60 publications) are summarized by stratification into five categories: (i) the whole-brain voxel-based analysis (VBA); (ii) the analysis of tract-based spatial statistics (TBSS); (iii) the ROI analysis; (iv) tractography; and (iv) the classification-based analysis. Since the DTI findings in the literature are mainly concerned with WM connectivity across multiple structures, classification here is not based on the structures, such as in Section 2. It is rather based on the methodology the WM connectivity and the fiber tracts through those structures are investigated with. These methods differ in how group differences are investigated by measuring DTI heterogeneity, e.g., the FA. The same four age stages, as in Section 2, i.e., infancy, childhood, adolescence, and adulthood, are considered separately for more in-depth presentation of the ASD findings.

#### 3.2.1. Whole-brain VBA

The brain images across subjects are spatially co-aligned in order to ensure that each individual voxel has the same anatomic location in all the subjects. Then the voxel-wise statistics are used to find areas of significant difference between the ASD and control patients. Due to its comprehensive examination, the whole-brain VBA overcomes problems of possible user bias in and insufficient prior knowledge for selecting the ROI to be analyzed. However, the VBA has drawbacks that are absent in the ROI analysis: high sensitivity to accuracy of aligning the images and low reliability of voxel-wise statistical decisions. In spite of these drawbacks, the VBA is still widely used due to its simplicity and ability to explore the whole brain.

##### 3.2.1.1. Childhood

Relationships between communication, social interaction, and repetitive behaviorial impairments have been investigated in Cheung et al. (2009) by the VBA of FA data for each group of subjects. For the ASD group, the bilateral prefrontal and temporal regions had reduced FA values, as well as lower FA along the frontal striatio-temporal pathways or more posterior brain pathways were associated with communication and social reciprocity impairments or repetitive behaviors, respectively. In a multi-modality study Ke et al. (2009), the WM abnormalities were investigated in HFA Chinese children, and the VBA showed that the WM density decreases in the right frontal lobe, left parietal lobe and right anterior cingulate. In addition, the FA values were lower in the frontal lobe and left temporal lobe. Combining the VBA and RBV of the DTI has showed consistent WM abnormalities in the HFA patients. Children with ASD and their unaffected siblings have been compared with the control group in Barnea-Goraly et al. (2010). The ASD and sibling groups had the prevalent reduced FA and λ_||_ values in the frontal, parietal, and temporal lobes, especially, in the regions related to social cognition. However, no significant relationships between the WM measurements in the ASD and sibling groups have been reported. To what extent disconnectivity of networks, which are important for social communication, relates to behavioral impairments in children with ASD was investigated in Poustka et al. (2012). The VBA indicated the decreased FA values in the uncinate fasciculus and right superior longitudinal fasciculus. The additional analysis revealed a negative correlation between the FA values of the affected fiber tracts and the ASD symptoms.

##### 3.2.1.2. Adolescence

An early study Barnea-Goraly et al. (2004) of the WM abnormalities performed the VBA on a small group of male HFA children and adolescents. It has found low FA values in various brain regions, mainly, in the WM adjacent to the ventromedial prefrontal cortices, in the anterior cingulate gyri, and in the temporoparietal junctions. A similar, but larger study Keller et al. (2007), has investigated the WM abnormalities in a large male HFA group aged from 10 to 35 years. The HFA subjects had lower FA values near the corpus callosum and in the right retrolenticular portion of the internal capsule. A new tissue-specific, smoothing-compensated (T-SPOON) VBA introduced in Lee et al. (2009) minimizes effects of partial volume averaging and image smoothing by applying a regional mask with the same smoothing parameters. Compared to the conventional VBA, results of the T-SPOON on a large group of the ASD subjects and corresponding controls were more consistent with the FA obtained by analyzing the corpus callosum and temporal lobe ROIs. The VBA of the WM of a small group of HFA and matched controls in Noriuchi et al. (2010) has shown the reduced FA and λ_⊥_ in various ROIs in the brain, including the left dorsolateral prefrontal cortex, cingulum bundle, arcuate fasciculus, and superior longitudinal fasciculus. Social impairment scores correlated negatively with the FA of the left dorsolateral prefrontal cortex, suggesting that the WM in cortical regions is vital for the ASD patients development. The WM integrity of the major fiber tracts connecting the amygdala, fusiform face area, and superior temporal sulcus in the ASD subjects was explored in Jou et al. (2011b) by the FA-based VBA. The ASD subjects had reduced FA values in the investigated WM tracts, including inferior longitudinal fasciculus, inferior fronto-occipital fasciculus, superior longitudinal fasciculus, corpus callosum, and cingulum bundle. A multi-modality study Groen et al. (2011) used the T1 MRI to study the brain volumetrics and the DTI to investigate both the WM and GM integrity in a limited group of HFA adolescents. There were no significant volumetric GM or WM differences between the two groups.

##### 3.2.1.3. Adulthood

The ASP adults, examined in Bloemen et al. (2010) for the WM integrity of the whole brain, have shown lower FA of 13 WM clusters in the internal capsule, frontal, temporal, parietal, and occipital lobes, as well as the cingulum and corpus callosum. Studying the WM abnormalities on a small group of young LFA men Pardini et al. (2012) indicated a positive correlation between the FA in the uncinate fasciculus and clinical improvement, precocity, and intervention duration.

#### 3.2.2. Tract-based spatial statistics (TBSS)

To overcome some shortcomings of the traditional VBA, the recent DTI-optimized analysis of TBSS Smith et al. (2006) uses a non-linear registration to a common target to co-align the subjects' FA maps. Following the alignment, a mean FA skeleton is built and thresholded to exclude areas of high inter-subject variability. Based on the corresponding FA values, each aligned subject's FA map is projected onto the skeleton to facilitate collecting standard voxel-wise FA statistics across all the subjects. Generally, such tract-based analysis is more accurate than the VBA, but requires a sophisticated data projection method for better results. It also does not handle partial volume effects, is sensitive to motion distortions, has high computational complexity, and may fail if the tracks change much at junctions or due to apparent pathologies.

##### 3.2.2.1. Childhood

The deterministic tractography and TBSS were combined in Kumar et al. (2010) to investigate the corpus callosum region, including the uncinate fasciculus, in young ASD children aged 5 years on average. The ASD manifests itself in lower FA, higher MD, larger number of streamlines and voxels, and longer streamlines. There were also ASD-related macrostructural changes in the uncinate fasciculus correlate with the popular symptomatic scores of the GARS (Gilliam autism rating scale). The TBSS, VOI, and tractography have been used also in Weinstein et al. (2011) to investigate the WM abnormalities of very young ASD children in several clusters within the genu and body of the corpus callosum, left superior longitudinal fasciculus, and right and left cingulum. The FA increased in these regions as a consequence of the decreased radial diffusivity, λ_⊥_. The tractography revealed that increased FA was concentrated in the mid-body of the corpus callosum and in the left cingulum.

To study the integrity of the thalamic radiation of older ASD children in Cheon et al. (2011), four DTI measurements, namely, FA, MD, λ_⊥_, and λ_||_, were examined in the anterior thalamic radiation, superior thalamic radiation, posterior thalamic radiation, corpus callosum, uncinate fasciculus, and inferior longitudinal fasciculus by combining the whole brain VBA analysis with the TBSS and ROI analyses. Anticipated WM abnormalities in the thalamo-frontal connections in the ASD children are indicated by the reduced FA and λ_||_ and Increased MD and λ_⊥_ across various brain regions. The TBSS, combined with a VBA were applied in Jou et al. (2011a) to a small group of older ASD and control children. The FA was reduced, especially, in the forceps minor, inferior fronto-occipital fasciculus, and superior longitudinal fasciculus. Regional distributions of differences between young ASD and control children were examined in Walker et al. (2012) using the TBSS and the whole-brain VBA. While the FA values were reduced in various brain regions, the increased MD was found only in the posterior ones. These small (1–2%) regional differences between both groups were accompanied by distinct regional differences in imaging artifacts. They also demonstrated vulnerability of the between-group differences to such artifacts, which may cause errors in biological inferences. The TBSS and deterministic tractography were also used in Billeci et al. (2012) to analyze young ASD children with and without mental retardation. The statistics have detected a widespread FA increase in major WM pathways, and the tractography showed increased FA and fiber length in the cingulum and corpus callosum. Moreover, the MD increase was correlated with expressive language functioning in the indirect segments of the right arcuate and left cingulum.

##### 3.2.2.2. Adolescence

Examining differences in the WM integrity between the HFA and control subjects and its association with pictorial reasoning under various linguistic levels in Sahyoun et al. (2010a) indicated that visuospatial reasoning performance relates to the FA of the peripheral parietal and superior precentral WM in the HFA subjects, but the superior longitudinal fasciculus, callosal, and frontal WM in the controls. The whole-brain VBA followed by analyzing the TBSS in Ameis et al. (2011) evaluated the WM in a group of ASD and control children and adolescents. The VBA has shown the increased MD and radial diffusivity (λ_⊥_) in the ASD children, but not the adolescents in the frontal WM, and the statistics analysis has revealed alterations in the right uncinate fasciculus. The right inferior longitudinal fasciculus in the ASD case may indicate a disrupted fronto-temporal-occipital circuit playing a significant role in social and emotional processing. The TBSS was analyzed in Shukla et al. (2011b) to examine short- and long-distance WM tracts in the frontal, parietal, and temporal lobes of the ASD subjects and matched controls. The short-distance tracts had reduced FA in the ASD group, increased MD and λ_⊥_ in the frontal, temporal, and parietal lobes. The age and DTI measurements were correlated in the control, but not ASD group. As was suggested, these typical age-related correlations were absent due to altered maturation of the short-distance tracts in the ASD group.

The whole-brain VBA with TBSS in Shukla et al. (2011a) assessed the WM tracts in the ASD children and adolescents. The reduced FA and the higher MD and radial diffusivity have been found for the ASD subjects in the corpus callosum, anterior and posterior limbs of the internal capsule, inferior longitudinal fasciculus, inferior fronto-occipital fasciculus, superior longitudinal fasciculus, cingulum, anterior thalamic radiation, and corticospinal tract. Moreover, the age-dependent analysis has shown no maturational changes in the ASD subjects. The WM tracts of a relatively larger group of the adolescents were studied in Bode et al. (2011) by examining the TBSS after correcting the entire image, rather than a ROI, which is more typical. As was found in the ASD group, the FA has increased, specially in the right inferior fronto-occipital fasciculus and affected visual perception. It suggests an abnormal information flow between the insular salience processing areas and occipital visual areas of the ASD patients.

##### 3.2.2.3. Adulthood

A recent fMRI and DTI study Kana et al. (2014) examined causal attribution in the ASD and confirmed the relationship for the temporo-parietal junction that exists in the theory of mind. The response to intentional causality showed lower activation of the temporo-parietal junction in the fMRI of the ASD adults, and the analysis of the tract-based spatial DTI statistics revealed reduced FA in the temporal lobe. The tract-based statistics were used also in a more recent investigation Perkins et al. (2014) of the WM integrity in a small group of the ASD adults and their matched controls. The ASD subjects showed significantly decreased FA and high radial diffusivity, λ_⊥_, values in the left hemisphere, mainly, in the thalamic and fronto-parietal pathways. Moreover, the WM disturbance was higher in the left hemisphere.

#### 3.2.3. ROI analysis

These analyses depend on prior assumptions about certain brain regions that might be impaired in the ASD subjects. The regions are extracted manually, semi-automatically (using delineation protocols), or automatically. Manual extraction is time-consuming and suffers from user variability, whereas automatic and semi-automatic region segmentation are affected by registration errors, as in the VBA. The fact that the VOI are grouped in a predefined way is the main advantage of analyzing the ROI, as the total number of multiple comparisons is much smaller than in the VBA, statistical decisions become more accurate. However, the ROI analysis cannot infer conclusions about microstructural properties of the WM tracts. It is impractical for examining every brain region, especially for large groups, and has limited accuracy because of low resolution and relatively thick slices of the DTI.

##### 3.2.3.1. Infancy

Microstructural WM differentiation between the ASD and control groups of infants and very young children was examined in Bashat et al. (2007). The fact that the left hemisphere's frontal lobe of the ASD subjects had predominately increased FA and probability, as well as reduced displacement supports the previous findings indicating an abnormal brain overgrowth in very young children.

##### 3.2.3.2. Childhood

The reduced FA of various cerebral WM tracts, which was found in Brito et al. (2009), suggests the ASD correlates with reduced connectivity in the corpus callosum, internal capsule, and superior and middle cerebellar peduncles. The ROI analysis of the cerebellar outflow and inflow pathways in Sivaswamy et al. (2010) has shown the bilaterally decreased MD in the superior cerebellar peduncles, together with the asymmetric FA of the middle cerebellar peduncle and the inferior cerebellar peduncle in the ASD patients. The effect of self-injurious behavior on cortical development of the ASD children was studied in Duerden et al. (2014) by using both T1 MRI and DTI scans. According to the sMRI analysis, both the thickness of the right superior parietal lobule and bilateral primary somatosensory cortices and the volume of the left ventroposterior nucleus of the thalamus correlate with the self-injury scores. The atlas-based ROI analysis has revealed that children engaged in self-injury had significantly decreased FA and increased MD in the the left posterior limb of the internal capsule, as well as increased radial diffusivity, λ_⊥_, in the bilateral posterior limbs of the internal capsule and corona radiate. Recently, an atlas-based ROI analysis Peterson et al. (2015) was used to investigate abnormalities in various left and right hemispheric WM regions of the HFA children. Their significantly increased MD of the outer-zone cortical left hemisphere WM suggested hypomyelination and increased short-range cortico-cortical connections caused by the early WM overgrowth.

##### 3.2.3.3. Adolescence

The entire corpus callosum and its subregions (genu, body and splenium) were explored for a large group of the HFA subjects and matched controls in Alexander et al. (2007) using the DTI (FA, MD, λ_⊥_, and λ_⊥_) and RBV. The low-IQ HFA subgroup differed by small corpus callosum volumes, increased MD, decreased FA, and increased λ_⊥_. The decreased FA and increased MD and λ_⊥_ in the superior temporal gyrus and temporal stem in the HFA subjects of the same group have been reported in Lee et al. (2007). Analyzing the WM over the whole brain and in several ROIs in Shukla et al. (2010) has detected decreased FA and increased λ_⊥_ in both the whole brain and corpus callosum; increased MD for the whole brain and the anterior and posterior limbs of the internal capsule; decreased λ_||_ in the corpus callossum body, and decreased FA in the middle cerebellar peduncle of the HFA subjects.

##### 3.2.3.4. Adulthood

The fMRI and DTI have been used in Thakkar et al. (2008) to determine whether the structure and function of the anterior cingulate cortex relate to a repetitive behavior in the ASD. The ASD subjects showed an increased rostral anterior cingulate cortex activation to both correct and erroneous responses and had reduced FA in the WM underlying the anterior cingulate cortex. These results correlate also with ratings of the rigid, repetitive behavior. A multi-modality study Beacher et al. (2012) used the VBM and ROI analysis to estimate the GM and WM volumes from the sMRI and evaluate the main WM tracts in the DTI, respectively, for the ASP adults. The total WM volume, regional GM volume in the right parietal operculum, and FA in the body of the corpus callosum, cingulum, and cerebellum suggested a correlation between the diagnosis and subject's gender. These findings confirmed the importance of understanding the sex-specific brain differentiation in the ASD.

#### 3.2.4. Tractography

Tractography reconstructs virtual 3D trajectories of the WM tracts to define the ROIs required for examining several such tracts simultaneously and characterizes macrostructural tract properties with additional DTI measurements. The deterministic tractography reconstructs the WM tracts and measures their lengths, densities, or volumes from a number of streamlines propagated between voxels with similar diffusion properties. But it provides no uncertainty of the reconstructed tracks due to noise or insufficient spatial resolution, whereas the probabilistic tractography not only estimates the fiber tracts, but also measures their uncertainty. However, branching and false-positive tracts affect the tractography accuracy in defining the ROIs, specifically at the ends of the reconstructed tracts. Also, the DTI tractography fails if the DT is inadequate to describe a region. For example, the FA is significantly lower for complex WM fiber crossings. To meet these challenges, new diffusion models, scanning paradigms, and analysis methods are constantly being developed.

##### 3.2.4.1. Infancy

Data from the IBIS (infant brain imaging study) group have been used for a recent longitudinal DTI analysis Wolff et al. (2012) of how the WM fiber tract is organized from 6 to 24 months in high-risk siblings' infants, who developed the ASD by 24 months. The FA and axial (λ_||_) and radial (λ_⊥_) diffusivity measured for the DTI were investigated to characterize microstructural properties of the WM fiber tracts. The FA for the infants who developed the ASD differed significantly from those who did not develop it in 12 out of the 15 fiber tracts investigated, including the fornix, uncinate fasciculus, and inferior longitudinal fasciculus. For most of the investigated ASD infants, the FA of the developing fiber tracts was higher at 6 months, had no differences at 12 months, and decreased at 24 months of age. This study provided evidence for the altered brain growth of the WM pathways related to manifesting autistic symptoms for the first year of life, thus confirming the critical importance of the longitudinal studies in revealing the age-related brain and behavior changes underlying the neurodevelopmental disorders. The DTI tractography in Elison et al. (2014) used the same IBIS group, but focused on determining whether specific oculomotor functioning and visual orienting patterns characterize 7-month-old infants diagnosed with the ASD at 24 months and detecting neural associates of their behaviors. The measurements included an average saccadic reaction time in a visually guided saccade procedure and the radial diffusivity, λ_⊥_, of corticospinal pathways and the splenium and genu of the corpus callosum fiber tracts. Strong association between the λ_⊥_ of the splenium of the corpus callosum and visual orienting latencies in low-risk infants has been found, but this correlation was missing in the infants having the ASD. These results confirm the potential of acquiring infant imaging groups in identifying early ASD markers, which is critical for early clinical intervention.

##### 3.2.4.2. Childhood

Whether the deterministic tractography of the DTI can detect the WM abnormalities in the frontal lobe and check for short range connectivity changes in the ASD children was investigated in Sundaram et al. (2008). The higher MD in the whole frontal lobe, as well as in long and short range association fibers in the ASD group have been reported. The FA was reduced in the ASD group for the short range, but not long range fibers, and the necessity of advanced DTI technology to re-examine the short range connectivity in ASD was indicated. The brain connectivity in the corpus callosum of the HFA patients was examined in Hong et al. (2011) by measuring both the DTI and T1 MRI. The corpus callosum volume and density extracted from the MRI were compared to the FA, MD, average fiber length, and fiber number obtained from the DTI by deterministic tractography. The decreased WM density in the anterior third of the corpus callosum, and the higher MD and lower fiber number in the anterior third transcallosal fiber tracts of the HFA subjects have been found. The frontal lobe association pathways in the ASD children were studied in Jeong et al. (2011) by analyzing the tract curvature, FA, λ_||_, and λ_⊥_. This study suggested that higher curvatures and λ_⊥_ in the parietotemporal junction of accurate fasciculus, frontotemporal junction of uncinate fasciculus, and the midline of the genu of the corpus callosum could be caused by larger attenuation of the thinner axons in the frontal lobe tracts of the ASD children.

The lack of the nonverbal ASD studies was addressed in Wan et al. (2012) by employing the probabilistic tractography to find language-related WM tracts (arcuate fasciculus). A small group of five nonverbal ASD children demonstrated the reversed arcuate fasciculus asymmetry. To what extent the ASD language ability is associated with the DTI measurements (FA and MD) of the language-related WM tracts (superior longitudinal fasciculus) and non-language-related WM tracts (corticospinal tracts) was investigated in Nagae et al. (2012) with the deterministic tractography. The obtained results have revealed the higher MD in the left hemisphere temporal portion of the superior longitudinal fasciculus fiber tracts of the ASD children with language impairment, as well as a significant negative correlation between the MD and language ability scores. The fMRI and probabilistic tractography of the DTI in Lai et al. (2012) examined the functional and structural organization of neural systems overlapping for language and music in the ASD children. The ASD children have demonstrated decreased functional responses to speech stimulation in the left inferior frontal gyrus and secondary auditory cortices in the left temporal lobe, as well as decreased FA of the left dorsal pathway and the decreased tensor norms in the ventral tract. All the findings indicated that speech and song processing functional systems are more responsive for the song than the speech of the ASD children.

Relations between the WM microstructure and developing the morphosyntax in a spoken narrative were examined in Mills et al. (2013) on a small group of the older HFA children and their matched controls. The HFA children showed abnormally increased MD in the right inferior longitudinal fasciculus. A positive correlation between the morphological accuracy and WM integrity in the right inferior longitudinal fasciculus was found within the HFA group. This study has shown that the HFA children rely on the ventral, rather than typical pathways in their daily use of real world language. Both the sMRI and probabilistic DTI tractography were used in Joseph et al. (2014) to investigate the GM and WM related to language ability in the young ASD children. Compared to the control group, the ASD children had decreased leftward asymmetry of volume and λ_⊥_ of the arcuate fasciculus, but no difference in the GM asymmetries.

##### 3.2.4.3. Adolescence

Examining the arcuate fasciculus in a group of ASD and control adolescent subjects with the probabilistic tractography Knaus et al. (2010) showed no group differences in FA. But atypical language laterality was more predominant in the ASD, than the control group. Advantages of volumetric DTI segmentation over tractography (its higher robustness to imaging noise, better compatibility with statistical analysis, and no region-to-region analysis) were exemplified in Fletcher et al. (2010) on extracting the WM tracts to study the same arcuate fasciculus on a group of the adolescent HFA and control subjects. The ASD group had increased MD and λ_⊥_, and decreased FA. The overlap between specific LI and ASD LI in a group of the adolescents and children has been studied in Verhoeven et al. (2012). The deterministic tractography was applied to extract the superior longitudinal fascicle, and no significant differences in the FA and MD have been revealed between the ASD LI participants and controls. However, the FA was significantly reduced in children with the specific LI compared to their controls. The diffusion tractography was used in Lo et al. (2011) to investigate three association fibers, such as the bilateral cingulum bundle, bilateral arcuate fasciculus, and bilateral uncinate fasciculus, as well as the callosal fiber tracts. High-resolution diffusion spectrum imaging (DSI) was applied to a small group of the HFA subjects and controls in order to assess generalized fractional anisotropy (FA) and asymmetry patterns in the targeted fiber tracts. The HFA adolescents showed no leftward asymmetry found in the controls, but had the significantly reduced generalized FA in the three callosal fibers. The deterministic tractography in Ameis et al. (2013) extracted the cingulum bundle from a larger group of children and adolescent HFA subjects and controls. Significant age group interaction for the FA and diffusivity (MD, λ_⊥_, and λ_||_), which is driven by reduced FA and increased diffusivity in the ASD groups, but not in the adolescent groups, has been revealed. The fMRI and DTI probabilistic tractography were used in Nair et al. (2013) to examine the integrity of thalamo-cortical connectivity in children and adolescents with the ASD. Increased MD and λ_⊥_ in the thalamo-cortical connections, and decreased functional connectivity in the thalamo-cortical circuitry, as well as a negative correlation between the fronto-thalamic FA and the social and total autism diagnostic observation schedule (ADOS) scores were reported. The cingulum bundle of a group of the ASD and control adolescents and young adults was investigated in Ikuta et al. (2014). By applying the probabilistic tractography, the low FA of the bilateral anterior cingulum bundle and negative correlation between this FA and the behavior rating inventory of executive function (BRIEF) scores were also identified.

##### 3.2.4.4. Adulthood

The deterministic tractography was used in Catani et al. (2008) to examine microstructural integrity of the intracerebellar pathways in the ASP adults. The study revealed reduced FA of the short intracerebellar fibers and right superior cerebellar output peduncle. It also showed negative correlation with the autism diagnostic interview (ADI) scores. The deterministic tractography in Conturo et al. (2008) reconstructed the WM tracts from the amygdala to the fusiform cortex, and the hippocampus to the fusiform cortex in the ASD and control adults. The ASD group has shown increased λ_⊥_ in the bilateral amygdala fusiform connections and left hemisphere hippocampus fusiform connections, as well as decreased λ_⊥_ in the right hippocampus-to-fusiform connections, being associated with the lower face recognition scores and performance IQ. The very first study Pugliese et al. (2009), which examined connections between socio-emotional structures in the adults with ASD using the deterministic tractography, reported an increased number of streamlines (tract volume) in the right cingulum bundle and inferior longitudinal fasciculus of the ASP adults in contrast to their reduced number of streamlines in the right uncinated fasciculus. Significant age-related differences between the autistic and control groups were found in the MD of the left uncinate fasciculus. The deterministic tractography was used also in Thomas et al. (2011) to extract the intra-hemispheric visual-association WM tracts for the HFA adults and detect their high numbers of streamlines in the intra-hemispheric fibers, mainly, in the left hemisphere, and a low number of streamlines in the minor forceps and the body of the corpus callossum. A multi-modality (sMRI and DTI) approach in Langen et al. (2012) has examined differences in the bulk striatum volume and fronto-striatal WM integrity and their relationship with repetitive behavior and inhibitory control of the ASD adults. Analyzing the MRI has shown a smaller WM volume, and the DTI tractography revealed reduced FA in the WM connecting putamen to the frontal cortical areas and increased MD of the WM tracts connecting the accumbens to the frontal cortex. The relationship between the inter-hemispheric connectivity and the brain overgrowth in the ASD adults has been tested in Lewis et al. (2013) with the probabilistic tractography. The estimated callosal fiber length measured the maximum brain size achieved during the development, and compared to the size and structure of the corpus callosum extracted from the T1-weighted MRI. In the ASD adults, the callosal fiber length correlated inversely with the corpus callosum size and positively with the radial diffusivity, λ_⊥_.

#### 3.2.5. Classification-based analysis

Since the advent of their basic concept in the mid-1980s, computer-aided diagnostic (CAD) systems remain in great demand among neuroradiologists Arimura et al. (2009). Most of the present CAD systems perform data preprocessing, feature extraction, and classification. Initial efforts to use features extracted from DTI to classify and diagnose ASD are detailed below.

##### 3.2.5.1. Childhood

Different DT coefficients from many areas across the brain were used in Ingalhalikar et al. (2011) to distinguish autistic children from controls. A high-dimensional nonlinear SVM has learned an underlying ASD pattern of numerous atlas-based regional DTI features, e.g., FA and MD, extracted from the various brain ROIs. According to the leave-one-out (LOO) cross validation, 84% specificity and 74% sensitivity were achieved in separating the autistic patients from the control ones.

##### 3.2.5.2. Adolescence

Shape representations of the WM tracts extracted from the DTI were utilized in Adluru et al. (2009) to distinguish between the autistic and control patients. These fiber bundles were seeded in the splenium of the corpus callosum, and the classification features were built using 3D shape context Belongie et al. (2002). The LOO cross-validation has resulted in an accuracy of 75%; both the specificity and sensitivity of 71%, and an average AUC of 0.765 (the area under the receiver operating characteristic curve). The DTI features of the ROI from the WM of superior temporal gyrus and temporal stem brain regions, which were assumed to have an important role in language, emotion, and social cognition development, were used in Lange et al. (2010) to study a large group of the HFA subjects and their matched controls. The extracted features included the FA, MD, λ_⊥_, λ_||_, normalized DT skewness, and hemispheric asymmetry index. Using the selected aforementioned six WM measurements, the system was able to separate the ASD subjects from the controls with 92% accuracy; 94% sensitivity, and 90% specificity.

### 3.3. Summary

Tables 5–8 summarize, in accordance with the life stage, basic current results of studying the ASD with the DTI.

**Table 5:** **DTI-based studying of the ASD at Infancy**.

**Rf**	**Autistic group**			**Control group**			**sMRI**	**Brain regions**	**Data analysis**	**Findings**
	**Age, mo**	**IQ**	**Size**	**Age, mo**	**IQ**	**Size**				
Bashat et al., 2007	22–40	–	7	4–23	–	18	1.5 T; 4.5 mm; 6000 b; 6 GD	CC genu and splenium; left posterior limb of IC; left EC; left forceps minor; left CST	ROI; *t*-test	ASD: Increased FA in CC genu and splenium; left hemisphere posterior limb IC, and left hemisphere EC; decreased FA left hemisphere CST; increased WM probability, and decreased displacement distribution
Wolff et al., 2012	t1: 6–7, t2: 12–13, t3: 23–25	t1: 67–115, t2: 69–111, t3: 65–109	24 HR	t1: 6–7, t2: 12–13, t3: 24–26	t1: 86–118, t2: 86–116, t3: 81–117	64	3 T; 2 mm; 1000 b; 25 GD	CC genu, body, and splenium; fornix; inferior longitudinal fasciculus; uncinate fasciculus; anterior thalamic radiation; IC anterior and posterior limbs	DT; ROI; RCLGCM; DTIprep; 3D Slicer; SAS 9.2	6-mo ASD: subjects: Increased FA in CC body, left hemisphere fornix, inferior longitudinal fasciculus, uncinate fasciculus, and posterior limb IC; 12-mo ASD subjects: no significant FA difference, except of decreased FA in left hemisphere anterior thalamic radiation; 24-mo ASD subjects: decreased FA in left hemisphere anterior limb IC and anterior thalamic radiation
Elison et al., 2014	7	–	16 HR	7	–	41 LR 40 HR	3 T; 2 mm; 1000 b; 25 GD	CC splenium and genu; left and right CSTs	DT; ROI; MANOVA; FiberViewer	ASD: Correlated λ_⊥_ in CC splenium and visual orienting latencies in LR infants; no correlation in HR infants

*Additional abbreviations: b, b-value (s/mm^2^); f, females; m, males; mo, month*;

*Rf, reference; T, MRI magnetic field strength (tesla); t1, t2, t3, time periods during longitudinal studies*;

*CC, corpus callossum; CST, cortico-spinal tract; DT, deterministic tractography; EC, external capsule*;

*GD, gradient directions; IC, internal capsule*;

*MANOVA, multivariate analysis of variances; RCLGCM, random coefficient linear growth curve model*;

*DTIprep, 3D Slicer, FiberViewer, SAS 9.2, data processing packages*.

**Table 6:** **DTI-based studying of the ASD at childhood**.

**Work**	**Autistic group**			**Control group**			**sMRI**	**Brain regions**	**Data analysis**	**Findings**
	**Age, y**	**IQ**	**Size**	**Age, y**	**IQ**	**Size**				
Sundaram et al., 2008	2–7	–	50 AD, ASP, NPDD	3–11	–	16	3 T; 3 mm; 1000 b; 6 GD	Frontal lobe's long-/short-range association fibers	DT; ROI; MANCOVA; DTI studio	ASD: Increased MD in short/long-range fibers and decreased FA in short-range fibers; insignificant negative correlation between FA and GARS AQ (social isolation subscale)
Brito et al., 2009	8–12	–	8 AD	8–12	–	8	1.5 T; 5 mm; 1000 b; 12 GD	Frontopontine and corticospinal tracts; frontal subcortical WM	ROI; ANOVA; EpiInfo	ASD: Decreased FA in the anterior CC, right CST, posterior limb of right and left ICs, left superior cerebellar peduncle, and right and left middle cerebellar peduncles
Cheung et al., 2009	7–12	78–122	13 AD	7–13	92–132	14	1.5 T; 5 mm; 1200 b; 25 GD	Left orbitofrontal cortex; precentral gyrus; bilateral frontal pole	VBA; GLM; Linear regression; SPM2; SPSS15.0	ASD: Decreased FA in prefrontal lobes, ventral and middle temporal lobe, and cerebellum; increased FA in superior longitudinal fasc and left occipital lobe; strongly correlated higher ADI-R scores and lower FA in prefrontal lobes and ventral temporal lobes; negatively correlated communication and social reciprocity impairments (ADI-A and ADI-B) throughout fronto-striato-temporal pathways and posterior CC
Ke et al. (2009)	6–11	82–120	12 HFA	7–12	82–118	10	1.5 T; 3 mm; 1000 b; 15 GD	Bilateral middle frontal gyrus; left inferior frontal gyrus; left superior temporal gyrus; right middle temporal gyrus; right frontal lobe	VBA; WM density; SPM5	ASD: Decreased WM density in right frontal lobe, left parietal lobe and right anterior cingulate; increased WM density in right frontal lobe, left parietal lobe and left cingulate gyrus; decreased FA in the frontal lobe and left temporal lobe; positively correlated CARS score and FA in right frontal lobe; no significant correlation between ADI-R scores and mean FA
Sivaswamy et al., 2010	2–9	–	27 AD, ASP, NPDD	2–9	–	16	3 T; 3 mm; 1000 b; 6 GD	Superior, middle, and inferior cerebellar peduncles tracts	ROI; ANCOVA; SPSS17.0	ASD: Increased MD of bilateral superior cerebellar peduncles; increased FA of right middle cerebellar peduncle; reversed FA asymmetry pattern in middle and inferior cerebellar peduncles
Kumar et al., 2010	2–9	–	32 AD, ASP, NPDD	2–9	–	16	3 T; 3 mm; 1000 b; 6 GD	Uncinate fasc.; inferior fronto-occipital fasc.; arcuate fasc.; CC; CST	TBSS; DT; ANOVA; DTI studio 2.40	ASD and DevI: Decreased FA in right uncinate fasc., right cingulum, and CC; increased MD in right arcuate fasc.; DevI: Decreased FA in bilateral fronto-occipital fasc
Barnea-Goraly et al., 2010	9–14	69–103	13 AD	8–12	107–123	11	1.5 T; 3 mm; 900 b; 6 GD	Medial prefrontal WM; frontal corona radiate; CC genu, anterior forceps, and body	VBA; FSL	ASD and siblings: Decreased FA and λ_||_ in multiple regions across frontal, temporal, and parietal lobes; no significant differences in WM structure; ASD: No significant correlation between ADOS and ADI-R subscale scores and FA or λ_||_
Weinstein et al., 2011	2–4	–	22 AD	2–5	–	32	1.5 T; 3 mm; 1000 b; 15 GD	CC genu and body; left superior longitudinal fasc.; right and left cingulum	TBSS; DT; MRI studio; FSL	ASD: Increased FA and decreased λ_⊥_ in CC genu and body, left superior longitudinal fasc., and bilateral cingulum
Hong et al., 2011	7–11	84–126	18 HFA	8–12	86–136	16	1.5 T; 2 mm; 1000 b; 15 GD	CC anterior third, anterior and posterior midbody, isthmus; and splenium	DT; ROI; WM density and volume; FSL; SPSS13.0	ASD: Decreased WM density in CC anterior third; increased MD and decreased fiber number in anterior third transcallosal fiber tracts; no significant correlation between DTI indices and CARS
Ingalhalikar et al., 2011	8–13	–	45 HFA	8–13	–	30	3 T; 2 mm; 1000 b; 30 GD	Middle occipital gyrus left; inferior occipital WM right; superior temporal WM right fornix	Classification; ROI; non-linear SVM	84% specificity and 74% sensitivity by LOO CV based on FA in right occipital regions, left superior longitudinal fasc., EC; IC; MD in right occipital gyrus and right temporal WM; correlated SRS/SCQ autism scores and classification results
Cheon et al., 2011	9–13	100–124	17 ASP; NPDD	8–12	103–125	17	1.5 T; 3 mm; 900 b; 30 GD	Anterior thalamic radiation; superior thalamic radiation; inferior longitudinal fasc	TBSS; ROI; ANOVA; DTIStudio; FSL; SPSS 11.5	ASD: Decreased FA and increased MD in right anterior thalamic radiation, CC, and left uncinate fasc.; decreased FA in left anterior thalamic radiation, and right and left inferior longitudinal fasc.; increased λ_⊥_ in right and left anterior thalamic radiation, CC, left uncinate fasc., and left inferior longitudinal fasc.; decreased λ_||_ in left inferior longitudinal fasc.; negatively correlated SRS and FA in right anterior thalamic radiations and right uncinate fasc
Jeong et al., 2011	3–7	–	32	4–8	–	14	3 T; 3 mm; 1000 b; 6 GD	Bilateral arcuate fasc.; bilateral uncinate fasc.; CC genu.	DT; ROI; TBSS; FSL; SPM	ASD: Increased curvature and λ_⊥_ and decreased FA in parietotemporal junction for arcuate fasc., frontotemporal junction for uncinate fasc., and midline of CC genu; positively correlated curvature and λ_⊥_ in all ROIs; Controls and ASD: Negatively correlated curvature and FA in all ROIs; Controls: Positively correlated curvature and λ_⊥_ only in CC genu
Jou et al., 2011a	7–15	–	15 AD	9–14	–	8	3 T; 2.5 mm; 30 GD	Inferior fronto-occipital fasc.; superior longitudinal fasc.; uncinate fasc.; cingulum.	TBSS; FSL; SPSS17.0	ASD: Decreased FA in numerous association, commissural, and projection tracts, especially, the forceps minor, fronto-occipital fasc., and superior longitudinal fasc.; no significant correlation between FA and SRS scores
Wan et al., 2012	6–8	–	5 LFA	9–14	–	5	3 T; 1.5 mm; 1000 b; 25 GD	Arcuate fasc	PT; ROI; FSL	Nonverbal ASD: no usual leftward pattern of arcuate fasc. asymmetry (actually the reversed pattern for four out of the five nonverbal subjects)
Walker et al., 2012	3–7	–	39 AD	3–7	–	39 AD	1.5 T; 2.5 mm; 1100 b; 50 GD	Cerebellum; CC genu, body and splenium; CSTs; pons	TBSS; RESTORE	ASD: Decreased FA in various WM regions; ncreased MD in posterior brain regions Significant age group interaction, indicating differences in FA and MD developmental trends between the two groups
Nagae et al., 2012	7–18 ASD/-LI: 7–15 ASD/+LI	–	18 ASD/-LI; 17 ASD/+LI	7-18	–	25	3T; 2mm; 1000 b; 30 GD	Superior longitudinal fasc.; temporal lobe component of the superior longitudinal fasc.; CSTs	DT; GLM; HRL; DTIStudio; SPSS 19.0	ASD/-LI: Increased MD in CSTs ASD/+LI: Increased MD in left hemisphere superior longitudinal fasc fiber tracts and temporal portion of superior longitudinal fasc ASD/±LI: Significant negative correlation between left hemisphere superior longitudinal fasc MD and clinical language ability assessment
Poustka et al., 2012	8–12	97–125	18 AD	8–12	98–128	18	1.5 T; 2 mm; 1000 b; 6 GD	Fornix; superior longitudinal fasc.; uncinate fasc.; CC	VBA; DT; SPM5; NeuroQlab	ASD: Decreased FA in bilateral uncinate fasc and right superior longitudinal fasc.; negative correlation between FA of affected fiber tracts and autism communication and interaction scores (ADI-R and ADOS); no significant altered GM or WM concentration after correction for multiple comparisons
Lai et al., 2012	7–15	–	16 LFA	7–16	–	18	1.5 T; 5 mm; 1000 b; 25 GD	Arcuate and inferior fronto-occipital fasc	PT; *t*-test FSL4.1	ASD: Decreased FA in left part of left dorsal pathway; significantly lower tensor norms for ventral tract
Billeci et al., 2012	4–8	47–93	22 AD, NPDD	3–8	90–108	10	1.5 T; 3 mm; 1000 b; 25 GD	CC; cingulum; arcuate fasc	TBSS; DT; ANCOVA; FSL; BET; FNIRT; eDTI; SPSS	ASD: Increased FA in major WM pathways, especially in CC, cingulum, arcuate fasc and IC; increased fiber length and FA in cingulum and CC, and increased MD in indirect segments of right arcuate and the cingulum; significant correlation between MD of arcuate fasc., CC, and cingulum and expressive language abilities
Mills et al., 2013	8–11	VIQ: 77–117	10 HFA	7–11	109–133	17	1.5 T; 2.5 mm; 1000 b; 51 GD	Superior longitudinal fasc.; temporal and partial subsections of superior longitudinal fasc.; inferior fronto-occipital fasc	DT; ROI; FreeSurfer; DTIStudio	ASD: Increased MD and λ_⊥_ in right inferior longitudinal fasc Positive correlation between higher FA and lower MD and λ_⊥_ in inferior longitudinal fasc produced more morphologically accurate language
Duerden et al., 2014	8–13	86–122	30 AD	8–13	100–128	30	1.5 T; 3 mm; 1000 b; 35 GD	Cingulum bundle; posterior limb of IC; corona radiata	ROI; TBSS; MANCOVA; Camino; FLIRT	ASD and self-injury subjects: Decreased FA and Increased MD in left posterior limb of ICs Positive correlation between self-injury and increased λ_⊥_ in bilateral posterior limbs of IC and corona radiata.
Joseph et al., 2014	4–6	VIQ: 73–119	20 AD	8–11	VIQ: 102–132	20	3 T; 2 mm; 1000 b; 15 GD	Arcuate fasc	PT; ANOVA; FSL4.1.2	Decreased leftward/increased rightward asymmetry of pars opercularis correlated with higher language ability and bilaterally increased FA and decreased λ_⊥_ of the arcuate fasc
Peterson et al., 2015	9–12	86–118	36 HFA	9–12	100–118	37	3 T; 2.2 mm; 800 b; 32 GD	Left and right hemispheric WM regions	ROI; ANCOVA; CATNAP; RESTORE; LDDMM	ASD: Increased MD throughout left hemisphere, particularly in outer-zone cortical WM Controls and ASD: Negative correlation between MD and age in left-hemisphere WM regions

*Additional abbreviations: b, b-value (s/mm^2^); f, females; fasc., fasciculus; m, males; Rf, reference*;

*T, MRI magnetic field strength (tesla); y, year*;

*ADI-R, autism diagnostic interview - revised; ADOS, autism diagnistic observation schedule*;

*ANCOVA, analysis of covariances; ANOVA, analysis of variances*;

*AQ, autistic quotient; CARS, childhood autism rating scale; CC, corpus callossum*;

*CV, cross-validation; CST, cortico-spinal tract; DevI, developmentally impaired*;

*DT, deterministic tractography; EC, external capsule; FWE, family-wise error*;

*GARS, Gilliam autism rating scale; GD, gradient directions; GLM, general linear model*;

*HRL, hierarchic regression model; IC, internal capsule*;

*LDDMM, large deformation diffeomorphic metric mapping; LOO, leave-one-out; ±LI, with/without LI*;

*MANCOVA, multivariate analysis of covariances; MANOVA, multivariate analysis of variances*;

*PT, probabilistic tractography*;

*RCLGCM, random coefficient linear growth curve model*;

*SCQ, social communication questionnaire; SLI, specific LI; SNR, signal-to-noise ratio*;

*SPM, statistical parametric mapping; SRS, social responsiveness scale; SVM, support vector machine*;

*TBSS, tract-based spatial statistics*;

*3D Slicer, BET, CATNAP, DTIprep, DTI studio*;

*eDTI, FiberViewer, FLIRT, FNIRT, FSL, MRI studio, NeuroQlab, RESTORE*;

*SAS, SPM, SPSS, data processing packages*.

**Table 7:** **DTI-based studying of the ASD at adolescence**.

**Work**	**Autistic group**			**Control group**			**sMRI**	**Brain regions**	**Data analysis**	**Findings**
	**Age, y**	**IQ**	**Size**	**Age, y**	**IQ**	**Size**				
Barnea-Goraly et al., 2004	11–18	89–113	7 HFA	10–16	98–116	9	3 T; 5 mm; 900 b; 6 GD	Right motor and premotor areas; temporoparietal junction; superior and middle temporal gyrus.	VBA; *t*-test SPM99	ASD: Decreased FA in ventromedial prefrontal cortices, anterior cingulate gyri, temporoparietal junctions, superior temporal sulcus bilaterally, temporal lobes approaching amygdala bilaterally, occipitotemporal tracts, and CC
Keller et al., 2007	12–26	87–117	34 HFA	13–25	101–119	31	3 T; 3 mm; 850 b; 6 GD	Posteriormidbody/isthmus of CC; left and right anterior corona radiata near CC genu	VBA; REVWMRA; SPM2	ASD: Decreased FA in areas within and near CC and in right retrolenticular IC portion Positively correlated group and linear age effect on FA resulted in posterior right IC limb
Alexander et al., 2007	9–24	95–121	43 AD, ASP, NPDD	10–22	101–125	34	3 T; 2 mm; 1000 b; 12 GD	CC genu, body and splenium	ROI; ANOVA; BCA; AIR; FSL	ASD subgroup: Decreased FA and increased MD and λ_⊥_ in CC; significantly lower performance IQ than for either ASP+NPDD or controls; positively correlated λ_⊥_ and processing speed during the performance IQ tests (ASD subjects with low FA and high MD seem to have the highest λ_⊥_ and slowest processing speeds)
Lee et al., 2007	9–23	95–121	43 AD, NPDD	10–22	101–125	34	3 T; 2 mm; 1000 b; 12 GD	ASD: Superior temporal gyrus; temporal stem	ROI; ANCOVA; FSL; SPSS14.0	ASD: In all regions, significantly decreased FA and significantly increased both MD and λ_⊥_; very little age-related FA changes Controls: Increased FA with age Controls+ASD: Decreased MD and λ_⊥_ with age
Lee et al., 2009	9–23	94–121	43 HFA, NPDD	10–22	101–125	34	3 T; 2.5 mm; 1000 b; 12 GD	CC; bilateral superior temporal gyrus; anterior cingulum bundle	VBA; T-SPOON; ANCOVA; AIR; FSL; SPSS 14.0	ASD: T-SPOON VBA is more consistent with ROI measurements in CC and temporal lobe regions; T-SPOON VBA found unobserved before in Lee et al. (2007) and significant focal CC body regions during FA vs. processing speed test
Adluru et al., 2009	9–23	–	41 HFA	10–22	–	32	2 mm	WM tracts through CC splenium	Classification; VBA; FSL	ASD vs. controls classification by WM tracts shapes: 75.3% accuracy, 71.9% specificity, and 0.7645 AUC
Noriuchi et al., 2010	11–17	86–100	7 HFA, NPDD	10–16	106–126	7	3 T; 2 mm; 800 b; 32 GD	Anterior cingulate cortex; left dorsolateral prefrontal cortex; right temporal pole; amygdala, superior longitudinal fasc.; occipitofrontal fasc.; mid- and left anterior CC	VBA, MVR; SPM2; SPSS 17.0	ASD: Reduced FA and λ_||_ in left dorsolateral prefrontal cortex, posterior superior temporal sulcus/temporo-parietal junction, right temporal pole, amygdala, superior longitudinal fasc. and occipitofrontal fasc.; negatively correlated FA of left dorsolateral prefrontal cortex and SRS scores
Lange et al., 2010	10–22	93–127	30 HFA	10–22	102–128	30	3 T; 2 mm; 1000 b; 12 GD	Superior temporal gyrus; temporal stem	Classification; ROI; QDA	ASD: Reversed hemispheric DT skewness asymmetry in superior temporal gyrus; increased DT skewness on right and decreased FA in left superior temporal stem; increased MD, λ_||_, and λ_⊥_ in right temporal stem; positively correlated FA of left superior temporal gyrus and composite Vineland score; negatively correlated FA of right temporal stem and performance IQ/language functioning ASD vs. controls classification by six WM measurements: 92% accuracy.; 94% sensitivity, and 90% specificity
Knaus et al., 2010	11–19	VIQ: 104	14 AD	11–19	VIQ: 119	20	3 T; 2 mm; 1000 b; 15 GD	Arcuate fasc	PT; ROI; ANOVA; FreeSurfer; FSL; BET	ASD vs. controls: More prevalent atypical language laterality; no significant group differences in FA, but subjects with more typical leftward lateralization had greater FA in arcuate fasc. across both groups
Fletcher et al., 2010	12–16	85–123	10 HFA	12–14	93–113	10	3 T; 2.5 mm; 1000 b; 12 GD	Arcuate fasc	ROI; LME models; ITK-SNAP	ASD: Increased MD and λ_⊥_ in the left arcuate fasc,; less lateralized MD and FA
Sahyoun et al., 2010b	11–15	89–113	12 HFA	11–16	97–115	12	3 T; 2 mm; 700 b; 60 GD	Inferior parietal and frontal sulcus; medial temporal gyrus; superior temporal sulcus; fusiform gyrus	PT; ROI; FSL	ASD: Decreased FA in pathways between inferior frontal sulcus and fusiform gyrus and in both hemispheres; secreased FA in pathways between Inferior frontal sulcus and medial temporal gyrus and in right hemisphere
Shukla et al., 2010	12–13	VIQ: 107–113	26 HFA	12–14	VIQ; 107–113	24	3 T; 5 mm; 2000 b; 15 GD	CC genu, body, and splenium; IC genu and anterior and posterior limbs; middle cerebellar peduncle	ROI; VBA; ANOVA; FSL; SPM5; SPSS 16.0	Decreased FA and Increased λ_⊥_ for whole-brain WM and all three segments of CC and IC; increased MD for whole brain and for IC anterior and posterior limbs; decreased λ_||_ in CC body and decreased FA in middle cerebellar peduncle
Sahyoun et al., 2010a	11–15	89–113	9 HFA	11–16	97–115	12	3 T; 2 mm; 700 b; 60 GD	Forceps minor; bilateral superior longitudinal fasc. and arcuate fasc.; bilateral inferior fronto-occipital fasc.; right cerebellar WM; bilateral uncinated fasc	TBSS; ANOVA; FSL; BET; IRTK	Controls: Increased FA in WM tracts connecting with frontal lobe: bilaterally within forceps minor, in left inferior fronto-occipital fasc adjacent to middle and inferior frontal gyri, left superior longitudinal fasc., and right posterior ASD: Increased FA bilaterally within uncinate fasc. in temporal lobe and in right superior longitudinal fasc. peripherally near middle frontal gyrus Group differences in correlations between task performance and FA of different brain areas
Jou et al., 2011b	9–17	46–116	10 AD, ASP, NPDD	10–18	87–123	10	1.5 T; 4 mm; 1000 b; 6 GD	Anterior radatiata and body of CC/cingulum; left superior longitudinal fasc./accurate fasc.; left inferior fronto-occipital fasc.; bilateral inferior longitudinal fasc	VBA; VOI; DT; BioImage Suite	ASD: Decreased FA in inferior longitudinal fasc./inferior fronto-occipital fasc., superior longitudinal fasc., and CC/cingulum
Ameis et al., 2011	9–15	79–119	19 AD, NPDD	8–16	86–116	16	3 T; 3 mm; 1250; 12 GD	Bilateral uncintate fasc.; bilateral inferior fronto-occipital fasc.; forceps minor and major	TBSS; VBA; HRM; FSL 4.1; ANALYZE 9.0; SPSS 15.0	ASD: Increased MD and λ_⊥_ in corticocortical and inter-hemispheric WM tracts, especially in children and within frontal lobe
Verhoeven et al., 2012	12–15	–	19 AD	9–11	12		3 T; 2.2 mm; 800 b; 45 GD	Superior longitudinal fasc	DT; ROI; GLM; ExploreDTI	No significant differences in FA and ADC between ASD-LI and control subjects; decreased FA in ASD-SLI subjects w.r.t. their controls
Shukla et al., 2011b	10–16	106–112	26 AD, ASP	10–16	VIQ: 105–111	24	3 T; 5 mm; 2000 b; 15 GD	Short and long distance WM tracts in frontal, parietal, and temporal lobes in both hemispheres	TBSS; ROI; ANOVA; FSL; FNIRT	ASD: Decreased FA and Increased MD and λ_⊥_ in short distance tracts in frontal lobe; increased MD and λ_⊥_ in short distance tracts in temporal and parietal lobes Significant positive correlations between age and FA and negative correlations between age and MD and λ_⊥_ in short-distance tracts in each lobe in controls, but only in frontal lobe of ASD subjects
Groen et al., 2011	12–16	80–116	17 HFA	14–17	96–114	25	1.5 T; 2.5 mm; 900 b; 30 GD	Superior longitudinal fasc./accurate fasc.; inferior longitudinal fasc.; left corona radiate	VBA; SPM5	ASD: Decreased FA in left and right superior and inferior longitudinal fasc. (but this effect is insignificant after adjusting for age and IQ); increased kurtosis of WM FA probability distribution; increased MD tin all brain regions and shifted GM and WM MD probability distributions; no difference in GM or WM volume
Lo et al., 2011	14–16	101–116	15 HFA	14–16	101–121	15	3 T; 2.7 mm; 4000 b; 102 GD	Bilateral cingulum bundle; arcuate fasc.; uncinate fasc	DSI; ROI; ODF; MARINA; SPM5; SPSS 13.0	ASD: Decreased GFA in the three callossal fiber tracts Consistent leftward asymmetry in three pairs of association fibers in controls, but not in ASD subjects
Shukla et al., 2011a	12–13	106–112	26 AD, ASP	12–14	VIQ: 105–111	24	3 T; 2.7 mm; 2000 b; 15 GD	Inferior longitudinal fasc.; inferior fronto-occipital fasc.; superior longitudinal fasc./accurate fasc.; cingulum	TBSS; TLCBT	ASD: Decreased FA and increased MD in IC anterior and posterior limbs, CC, inferior and superior longitudinal fasc., inferior fronto-occipital fasc., CST, cingulum, and anterior thalamic radiation Age was positively correlated with FA in clusters of significant group differences and negatively correlated with MD and λ_⊥_ in controls, but not in ASD
Bode et al., 2011	13–15	–	27 HFA	13–16	–	26	3 T; 3.5 mm; 1000 b; 40 GD	Optic radiation; right inferior fronto-occipital fasc	TBSS; BET; FNIRT	ASD: Increased FA and decreased λ_⊥_ in the area containing clusters of optic radiation and right inferior fronto-occipital fasc.; no age-related correlations
Ameis et al., 2013	9–16	–	19 HFA	8–16	–	16	3 T; 3 mm; 1250; 12 GD	Cingulum bundle	DT; Diffusion Toolkit; SPSS 20	ASD: Significant age-group interaction effects across FA, MD, λ_⊥_, and λ_||_ in cingulum bundle; increased MD, λ_⊥_, and λ_||_ and decreased FA in cingulum bundle of younger subjects
Nair et al., 2013	11–17	VIQ: 96–127	26	12–16	VIQ: 95–117	27	3 T; 2 mm; 1000 b; 61 GD	Thalamic connections with prefrontal, parietal-occipital, motor, somatosensory and temporal cortices	PT; FSL	ASD: Increased MD in tracts connecting thalamus with motor and somatosensory cortices bilaterally and with prefrontal ROI in right hemisphere; increased λ_⊥_ in thalamic tracts for motor ROI bilaterally and somatosensory ROI in left and prefrontal ROI in right hemisphere, with a further marginal increase for somatosensory ROI in right hemisphere; negatively correlated FA and social and total ADOS scores in fronto-thalamic tracts and temporo-thalamic connections in left hemisphere ASD and controls: No FA or tract volume differences
Ikuta et al., 2014	15–21	77–117	21 AD, ASP	15–21	98–122	21	3 T; 2.5 mm; 1000 b; 31 GD	Cingulum bundle; anterior thalamic radiation	PT; ROI; ANCOVA; FSL; FLIRT	ASD: Decreased FA within cingulum bundle Significant group-age interaction, such that ASD subjects show no typical age-associated FA increases observed among controls; significant negative correlation between cingulum FA and total BRIEF score in ASD subjects, but not controls; ngatively correlated cingulum FA and shifting subscale in ASD subjects, but not in controls; neither group effect, nor group–age interaction in anterior thalamic radiation FA; insignificantly correlated anterior thalamic radiation FA and BRIEF BRI scores

*Additional abbreviations: b, b value unit (s/mm^2^); f, females; fasc., fascilus; m, males; y, year*;

*vs., versus; w.r.t., with respect to; REVWMRA, Random-effects voxel-wise multiple regression analysis*.

*ADC, apparent diffusion coefficient; ADOS, autism diagnostic observation schedule*;

*AIR, automated image registration; ANCOVA, analysis of covariances; T, MRI magnetic field strength (tesla)*;

*ANOVA, analysis of variances; AUC, area under ROC curve*;

*BCA, bivariate correlation analysis; BRIEF, behavior rating inventory of executive function*;

*CC, corpus callossum; CST, cortico-spinal tract; DSI, diffusion spectrum imaging*;

*DT, deterministic tractography; GD, gradient directions*;

*GFA, generalized FA; GLM, general linear models; HRM, hierarchical regression model*;

*IC, internal caplsule; LME, linear mixed effect*;

*MANOVA, multivariate analysis of variances*;

*MVR, multivariate regression; ODF, orientation distribution function; PT, probabilistic tractography*;

*QDA, quadratic discriminant analysis; ROC, receiver operating characteristic*;

*SLI, specific LI; SRS, social responsiveness scale*;

*TBSS, tract based spatial statistics; TLCBT, tract labeling cluster-based thresholding*;

*3D Slicer, ANALYZE, BET, BioimageSuite, Diffusion Toolkit*,

*DSI Studio, DTIprep, ExploreDTI, FiberViewer, FLIRT, FNIRT, FreeSurfer, FSL, IRTK, ITK-SNAP, MARINA, PSL*,

*RESTORE, SAS, SPM, SPSS, T-SPOON, data processing packages*.

**Table 8:** **DTI-based studying of the ASD at adulthood**.

**Work**	**Autistic group**			**Control group**			**sMRI**	**Brain regions**	**Data analysis**	**Findings**
	**Age, y**	**IQ**	**Size**	**Age, y**	**IQ**	**Size**				
Catani et al., 2008	22–40	92–126	15 ASP	24–46	99–141	16	1.5 T; 2.5 mm	Inferior, middle, and superior cerebellar peduncles; intracerebellar fibers	DT; ROI; RMA; SPSS	ASD: Decreased FA in short intracerebellar fibers and right superior cerebellar (output) peduncle; negatively correlated FA of left superior cerebellar peduncle and ADI-R social scores ASD and control groups: no MD differences
Conturo et al., 2008	23–29	102–106	17 HFA	23–29	99–141	17	1.5 T; 2.5 mm; 7 GD	Hippocampo- and amygdalo-fusiform pathways	ROI; DT; JMP 7.0	ASD: Decreased λ_⊥_ in right hippocampo-fusiform pathway; increased λ_⊥_ and λ_||_ in left hippocampo-fusiform pathway and bilateral amygdalo-fusiform pathways; decreased across-fiber diffusivity related to poorer Benton face interpretation and performance IQ scores
Thakkar et al., 2008	19–41	VIQ: 112–136	12 ASP, NPDD	19–35	VIQ: 95–123	12	3 T; 2 mm; 700 b; 72 GD	Anterior cingulate cortex	ROI; FLIRT; FSL; TKregister2	ASD: Decreased FA in WM underlying rostra1 and dorsal anterior cingulate cortex bilaterally, dorsolateral prefrontal cortex, ventral prefrontal cortex, and intraparietal sulcus; increased FA in right insula; increased activation on correct trials and reduced FA in rostra1 anterior cingulate cortex WM, which is associated with higher ratings of repetitive ADI behavior
Pugliese et al., 2009	11–35	93–117	12 ASP	15–35	105–137	42	2.5 mm; 1300 b	Inferior longitudinal fasc.; inferior frontal occipital fasc.; uncinate fasc.; cingulum; fornix	DT; ROI; GLM; Z-observation analysis; SPSS	No significant between-group differences in FA and MD ASD: Increased number of streamlines in right and left cingulum, and right and left inferior longitudinal fasc.; decreased number of streamlines in right uncinate fasc Significant age-related differences in MD and number of streamlines, but not FA within each group; significant age-related between-group MD differences in left uncinate fasc,
Bloemen et al., 2010	29–49	94–126	13 ASP	27–47	101–129	13	3 T; 2.5 mm; 1300 b; 64 GD	Inferior fronto-occipital fasc.; minor and major forceps; anterior and posterior corona radiata; bilateral anterior thalamic radiation	VBA; ANCOVA; permutation-based testing; SPM2; SPSS 12.0; XBAM	ASD: Reduced FA and increased λ_⊥_ over large brain areas; decreased MD in brain-stem cluster
Beacher et al., 2012	22–42 m; 25–39 f	–	28 ASP	20–36 m; 24–40 f	–	30	1.5 T; 2.6 mm; 1000 b; 64 GD	CC; cingulum bundle; CST; cerebellum	ROI; ANOVA; Diffusion Toolkit	Significant sex–diagnosis interactions in total WM volume, regional GM volume in right parietal operculum, and FA in body of CC, cingulum, and corona radiata *Post-hoc* comparisons: increased FA in male controls w.r.t. females, but no sex difference in ASD subjects
Thomas et al., 2011	19–39	97–117	12 HFA	18–27	102–122	18	1.5 T; 3 mm; 850 b; 6 GD	Inferior longitudinal and fronto-occipital fasc.; uncinate fasc.; 3 sub-portions of major inter-hemispheric fiber tract; CC	DT; ROI; ANOVA; DTIstudio	ASD: Increased WM volume of intra-hemispheric fibers, particularly, in left hemisphere, and decreased WM volume of minor forceps and CC body; negatively correlated minor forceps WM volume and ADI-R repetitive and stereotypical behavior scores; no group FA differences
Langen et al., 2012	20–32	92–122	21 AD	22–34	96–124	22	3 T; 2.4 mm; 1300 b; 32 GD	Fronto-striatal WM tracts	DT; ROI; GLM; MANOVA; SROC; ExploreDTI; FLIRT; TrackVis; SPSS	Decreased total brain WM volume and FA and increased MD of the tracts connecting putamen and accumbens to frontal cortical areas in ASD subjects ASD subjects had worse performance than controls on a go/nogo task No significant relationship between differences in FA and on ADI-R or ADOS scores in ASD subjects
Pardini et al., 2012	21–23	47–51	22 LFA	–	–	–	3 T; 2 mm; 1000 b; 33 GD	uncinate fasciculus	VBA; FSL; SPM5; SPSS16	Independent from symptoms' severity and IQ at therapy onset and from subject's age at time of MRI scanning and significant correlation between clinical improvement and FA of two WM clusters in uncinate fasc.; independent of symptoms severity and IQ scores and significant correlation between increasing uncinate fasc. structural organization and clinical improvement, precocity, and intervention length; more significant clinical improvement and higher uncinate fasc. FA for highly therapy-adherent subjects w.r.t. moderately adherent ones
Lewis et al., 2013	22–42	68–108 VIQ	20 AD	22–42	–	22	3 T; 3 mm; 1400 b; 15 GD	CC	PT; ROI; RFTCS; CIVET	ASD: Negatively correlated CC fiber length, adjusted for intracranial volume, and CC size; positively correlated adjusted CC fiber length and λ_⊥_
Kana et al., 2014	20–22	102–112	8 AD	21–23	110–114	13	3 T; 3 mm; 1000 b; 12 GD	Posterior CC midbody; corona radiata; WM underlying right middle/superior temporal lobe	TBSS; ANOVA; FSL	ASD: Decreased FA in WM underlying temporal lobe; decreased functional connectivity participants in ToM-related areas and ventral premotor areas; no relationship between DTI and fMRI results
Perkins et al., 2014	15–25	–	12 HFA, ASP	16–22	–	12	3 T; 2.5 mm; 1000 b; 25 GD	Superior longitudinal fascicles; accurate fasc.; cingulum bundle; CC genu, splenium, and body; IC anterior and posterior limb	TBSS; SA; GLM; FSL	ASD: Decreased FA and Increased λ_⊥_ in left hemisphere, predominantly thalamic and fronto-parietal pathways; significantly increased WM disturbance in left w.r.t. right hemisphere, according to symmetry analysis

*Additional abbreviations: b, b value unit (s/mm^2^); f, females; fasc., fascilus; m, males; y, year*;

T, MRI magnetic field strength (tesla); w.r.t., with respect to

*ADI -R, autism diagnostic interview – revised; ANCOVA, analysis of covariances*;

*ANOVA, analysis of variances; CC, corpus callossum; CST, cortico-spinal tract*;

*DT, deterministic tractography; GD, gradient directions; GLM, general linear models*;

*IC, internal caplsule; MANOVA, multivariate analysis of variances*;

*PT, probabilistic tractography; RMA, repeated measures analysis*;

*SROC, Spearman's rank-order correlation;; ToM, theory of mind*;

*CIVET, Diffusion Toolkit, DTIstudio, ExploreDTI, FiberViewer, FLIRT, FSL, JMP7, RFTCS, RMA, SPM, SPSS*,

*Tkregister2, TrackVis, XBAM, data processing packages*.

## 4. Discussion

The overviewed results of the widespread investigation of the sMRI and DTI modalities in ASD diagnostics outline promising directions of the future work.

###  

####  

##### Diagnosing ASD with sMRI

Obviously, from the sMRI investigations completed to date, it is clear that the brain of an autistic child is growing abnormally through early childhood. However, a consistent pathology amongst the autistic children is yet to be identified, and few, if any, studies include patients below 2 years of age. Nonetheless, early brain overgrowth does appear to be a consistent and repetitive ASD feature, but this feature is heterogeneous and not all autistic children have an enlarged cerebral volume. Moreover, due to a common misconception of gender bias in the ASD, most of the studies primarily or exclusively focused on males. Also, the statistical significance of the reported findings are hindered due to the small population of subjects considered.

Within the literature, the effects of age on various anatomical features such as, the dynamics of the amygdalae and cerebral volumes, tend to be contradictory. Furthermore, multiple cross-sectional studies examining brain stem size in ASD patients have shown inconsistent results, and no study to date specifically assesses how the brain stem develops with age. Also, studying the brain stem should become volumetric and focus on selecting proper planes of cut, because most of the current studies have transected the brain stem and provided only area measurements. As a result, additional longitudinal studies are needed before any conclusions can be drawn on the role of brainstem development in autism.

Deciphering the role of different brain regions and networks in autism may benefit significantly from using multimodality approaches, which combine various resting state, task-evoked, and structural MRI measurements and rely on advanced data acquisition and analysis.

The utilization of MRI-based methods to study brain development in larger populations will likely begin to address heterogeneity in ASD patients and identify distinct autism subtypes, each with a specific associated neuroanatomical phenotype. However, it is anticipated that the maximum scientific benefit will be achieved from conducting longitudinal studies in younger populations with additional characterization of the underlying genetic abnormalities, along with other potential biomarkers, driving the overgrowth and onset of autistic symptoms at a critical time in the developmental process. Hence, it is of great significance to establish more effective biomarkers in order to facilitate diagnostics in patients before their conventional ASD symptoms become evident.

Future studies that include more than two time points of MRI data would be particularly helpful in the construction and comparison of ASD and normal brain development patterns. Yet, another promising method for understanding the relationship between brain development and ASD is to statistically analyze local 3D shapes and surfaces of the primary components of the brain and correlate these results to the common behavioral scores, such as the social responsiveness scale (SBS). However, correlating the results will be challenging, since such scales were developed as screening tools to qualitatively describe ASD severity, rather than to quantify the health deficits.

##### Diagnosing ASD with DTI

The VBA, applied mostly to small and predominantly adolescent groups, has shown decreased FA in the major WM tracts of patients with ASD. The usefulness of VBA of DTI remains in doubt because this method depends on the size of the smoothing kernel and hinders confident conclusions. The TBSS, optimizing the VBA of DTI and thus outperforming the traditional VBA, have been used in the majority of cases to examine the whole brain in older children and adolescents. Most of the larger studies have reported reduced FA and increased diffusivity in the ASD groups. Also, the ROI-based DTI studies of the older population (older children, adolescents, and adults) vs. their controls confirm the finding of decreased FA in various brain areas including the anterior cingulate, the superior temporal gyrus, and the temporal stem WM. However, since the ROIs target only specific components of particular WM tracts, conclusions cannot be drawn on the entire WM tract or other infrequently examined tracts. Nonetheless, studying the DTI of older children, adolescents, and adults using the VBA, TBSS, and ROI methods suggest that the ASD-related microstructural WM alterations differ depending on the age of the individuals studied.

Mostly, the deterministic, rather than the advanced probabilistic tractography is employed to study the DTI, and the bulk of these studies have focused on the WM abnormalities in the uncinate fasciculus, the arcuate fasciculus, the cingulum bundle, the inferior longitudinal fasciculus, and the inferior fronto-occipital fasciculus. The abnormalities include lower FA and higher diffusivity (primarily, in children and adolescents) and altered macrostructure (mainly, in adults). The only ASD-related longitudinal DTI study to date Wolff et al. (2012) confirmed that at least during the first 2 years of life, a number of WM tracts seem to be affected by a dynamic process occurring in the brains of children who develop the ASD.

##### General conclusions

Although a variety of MRI-based studies consisting of small groups of individuals, ranging widely in age, present diverse clinical ASD symptoms compared to controls, these studies have yielded valuable, yet varying results. Multi-site studies with large patient groups that comprehensively characterize the clinical, behavioral, and cognitive symptoms may be one of the most important strategies for increasing the probability for the discovery of new, effective biomarkers. Future studies of particular importance will track brain maturation from infancy to adulthood in individuals at high risk of developing ASD by using multi-modality macro-/microstructural and functional brain images. By studying a larger, non-gender biased population of patients, it is anticipated that the “false positive” rate of ASD diagnosis will decrease.

## Author contributions

MI: wrote the structural MRI survey part, and helped in DTI part. Reviewed the entire paper. RK: reviewed the paper, reshaped the outline and structure. MM: wrote the DTI part. AELT: reviewed the paper, helped collecting literature. MC: reviewed, emphasized the clinical aspect of the paper, edited the future directions section. GG: reviewed the engineering aspect of the paper, reshaped the paper, and extensively edited it. AELB: directed the paper, helped with the outline and the general structure.

### Conflict of interest statement

The authors declare that the research was conducted in the absence of any commercial or financial relationships that could be construed as a potential conflict of interest. The reviewer and handling Editor declared their shared affiliation, and the handling Editor states that the process nevertheless met the standards of a fair and objective review.
